# Responses of root system architecture to water stress at multiple levels: A meta-analysis of trials under controlled conditions

**DOI:** 10.3389/fpls.2022.1085409

**Published:** 2022-12-09

**Authors:** Xinyue Kou, Weihua Han, Jian Kang

**Affiliations:** ^1^ Institute of Environment and Sustainable Development in Agriculture, Chinese Academy of Agriculture Sciences, Beijing, China; ^2^ Interdisciplinary Plant Group, University of Missouri, Columbia, MO, United States; ^3^ Division of Plant Science and Technology, University of Missouri, Columbia, MO, United States

**Keywords:** root system architecture, water deficit, genes and QTLs, hormones, root length density (RLD) distribution

## Abstract

Plants are exposed to increasingly severe drought events and roots play vital roles in maintaining plant survival, growth, and reproduction. A large body of literature has investigated the adaptive responses of root traits in various plants to water stress and these studies have been reviewed in certain groups of plant species at a certain scale. Nevertheless, these responses have not been synthesized at multiple levels. This paper screened over 2000 literatures for studies of typical root traits including root growth angle, root depth, root length, root diameter, root dry weight, root-to-shoot ratio, root hair length and density and integrates their drought responses at genetic and morphological scales. The genes, quantitative trait loci (QTLs) and hormones that are involved in the regulation of drought response of the root traits were summarized. We then statistically analyzed the drought responses of root traits and discussed the underlying mechanisms. Moreover, we highlighted the drought response of 1-D and 2-D root length density (RLD) distribution in the soil profile. This paper will provide a framework for an integrated understanding of root adaptive responses to water deficit at multiple scales and such insights may provide a basis for selection and breeding of drought tolerant crop lines.

## Introduction

Root system underpins the development of terrestrial vegetation as it anchors plants in the soil and provides the main route through which plants acquire water and nutrient from the soil (Sebastian et al., 2016; Ma et al., 2018; Fromm, 2019; Gupta et al., 2020). Plants are routinely exposed to myriad environmental stresses threatening plant survival, growth, and reproduction in the natural ecosystems (Huang et al., 2014; Kramer-Walter et al., 2016) and affecting crop yield and quality (Maqbool et al., 2022) in the agricultural system (FAO, 2020). Drought is one of these stresses (Gupta et al., 2020) and root has evolutionarily become the first organ that senses the changes in soil moisture and adapts to them at morphological, anatomical, and molecular scales (Amtmann et al., 2022). Fresh water availability is projected to decline by 50% owing to climate change, whereas water demand for agriculture is expected to double by 2050 (Gupta et al., 2020). Overcoming the water challenge in agriculture is central to achieving Zero Hunger, one of the 17 goals proposed in the 2030 Agenda for Sustainable Development (FAO, 2020). Hence, producing high-yielding crops under water-limiting conditions, particularly in the dryland agricultural system, is required to ensure global food security. Despite the significant advances made in the understanding of adaptive mechanisms of above-ground parts under changing climate, such research in plant root systems has not received due attention (Li et al., 2021; Moran et al., 2017). Hence, root has become an important target for genetic selection and modification in an effort to enhance crop resilience and maintain yield and quality under water-limiting conditions (Fromm, 2019; Kamoshita et al., 2008). A better understanding of plant root systems has been widely recognized as a key component of the second green revolution (Lynch, 2007), especially in the regions with low-input agricultural systems (Villordon et al., 2014). In this case, the patterns of root growth and responses under water-limiting conditions are fundamental aspects regarding crop production especially in arid areas (White, 2019).

Root system architecture (RSA) is the spatial distribution of roots in the soil profile (Lynch, 1995; Koevoets et al., 2016; Pandey and Bennett, 2019), and it is primarily shaped by length, branching, angle, and thickness. RSA is characterized by a series of traits including rooting depth, root growth angle, root-to-shoot ratio, root diameter, root length density, root surface area, root volume, root distribution, and root tip frequency and root hair development (Germon et al., 2020; Siddiqui et al., 2021). Roots have developed the ability to change the RSA traits in response to water stress (‘plasticity’) (Fromm, 2019; Gupta et al., 2020; Siddiqui et al., 2021; Kang et al., 2022). Nevertheless, these traits are not equally sensitive to drought (de Vries et al., 2016; Stagnari et al., 2018). A review showed that drought decreased total root length and tip frequency, increased rooting depth and had no effect on root branching in tree species (Brunner et al., 2015). It is becoming increasingly evident that considerable inter- and intraspecies variations are present in the drought response of RSA (Guenni et al., 2002; Belachew et al., 2018; Berny-Miery et al., 2019; Fromm, 2019). Plasticity of plant traits are closely associated with drought tolerance which is generally evaluated as the capability of surviving and maintaining growth and yield under drought conditions (Volaire and Lelievre, 2001; Feller and Vaseva, 2014; Gao et al., 2015; Suseela et al., 2020). Similar to the plasticity of shoot traits that have a great impact on plant reproductive performance (Funk et al., 2021), root traits are also highly correlated with crop yield (Uga et al., 2013; Ogura et al., 2019) and yield stability (Sandhu et al., 2016) under drought conditions. Therefore, understanding how RSA is responding to water stress and regulated by genetic and metabolic mechanisms in plants has great importance on agricultural sense, which can be potentially manipulated for crop improvement.

A number of recent reviews have focused on how root traits respond to drought at a certain scale (s) (Wasson et al., 2012; Comas et al., 2013; Lynch, 2013; Khan et al., 2016; Valliyodan et al., 2017; Lynch, 2018; Ye et al., 2018; Kim et al., 2020; Siddiqui et al., 2021). A meta-analysis study of root traits, based on 128 published studies under field conditions, has shown that drought significantly decreased root length and root length density, while it increased root diameter and root-to-shoot biomass ratio (Zhou et al., 2018). A whole-genome meta-analysis was performed to find out candidate genes and genomic regions involved in controlling RSA traits under well-watered and drought stress conditions in rice (Daryani et al., 2022) and other major cereal crops including maize (Guo et al., 2018), bread wheat (Darzi-Ramandi et al., 2017; Soriano and Alvaro, 2019; Bilgrami et al., 2020), and durum wheat (Iannucci et al., 2017). Specifically, plant hormones are known to play critical roles in the molecular regulation of RSA traits under drought and the progress has been reviewed by Ranjan et al. (2022). Beyond these factors, root distribution in the soil, generally described by the distribution of root length density (RLD), at different dimensional scales determines the efficiency of water and nutrient uptake (Ahmadi et al., 2011; Ahmadi et al., 2014; Thidar et al., 2020). A few studies have shown that plants adjusted root distribution under drought conditions to access water available in different soil layers (Jongrungklang et al., 2012; Fitters et al., 2017; Morris et al, 2017; Song et al., 2020; Shabbir et al., 2021). However, up-to-date knowledge on responses of RSA in a wide range of plant species at multiple scales has not been well analyzed.

**Figure 1:**
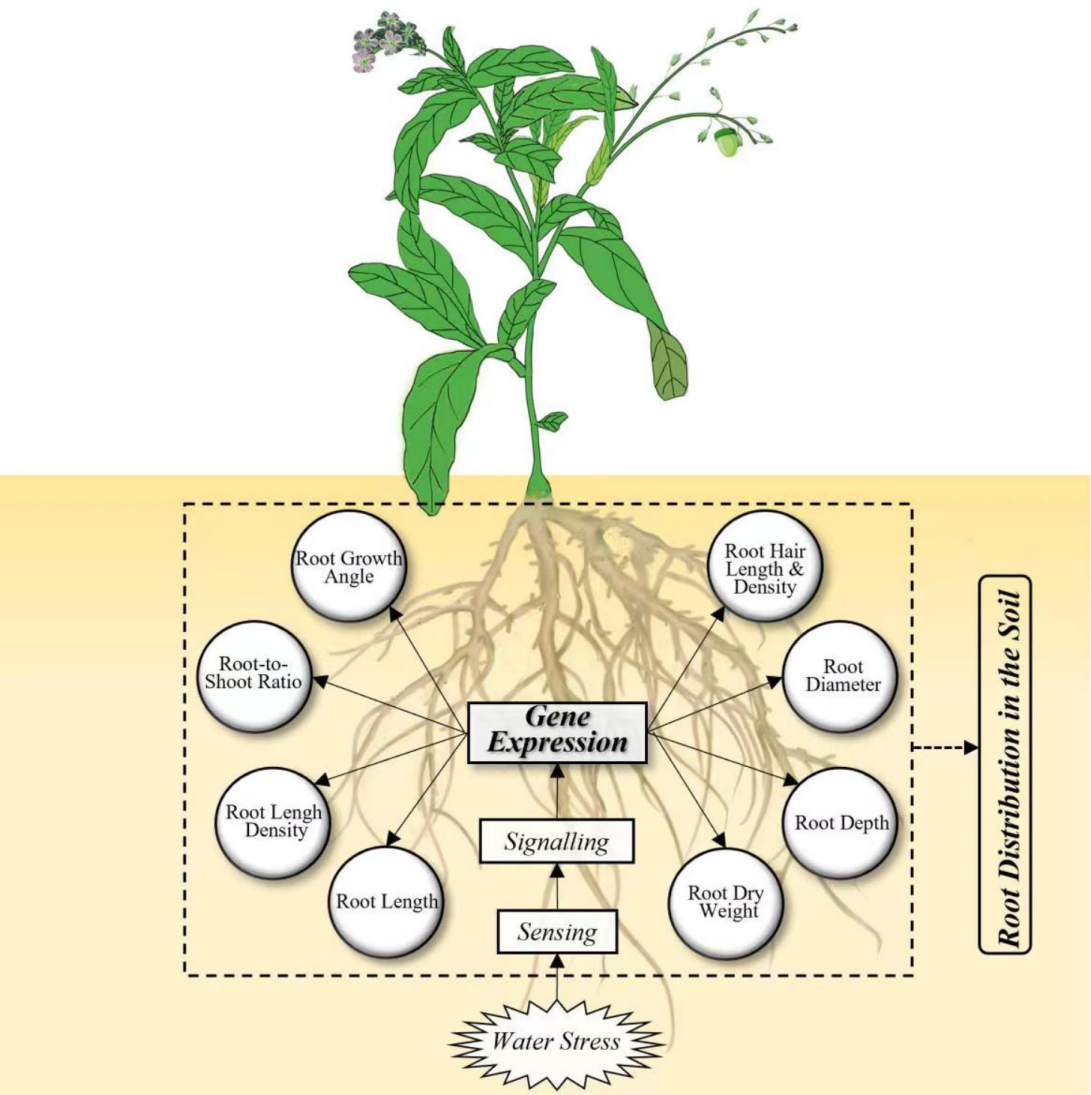
Framework integrating the root responses to water stress at genetic and morphological levels.

Therefore, this paper synthesizes up-to-date knowledge on RSA drought responses in a wide range of plant species at multiple scales (**Figure 1**). We briefly summarized the genes and QTLs and hormones that are involved in the drought response of root traits. We then statistically analyzed the drought responses of typical root traits under controlled manipulative experiments and discussed the underlying mechanisms. We finally generalized the responses of 1-D root length density distribution to drought and well-watered conditions in the soil profile. Meanwhile, we discussed the effects of water deficit on 2-D root length density distribution and 3-D RSA and its regulation mechanism.

## Methods

### Literature search and data selection

We screened the literature using different combinations of key words that indicate water availability (“water deficit”, “water stress”, “water deficiency”, “water shortage”, or “drought”) and describe the root traits (“root growth angle”, “root depth”, “root length”, “root diameter”, “root dry weight”, “root-to-shoot ratio”, “root length density”, and “root hair”) in Web of Science (https://www.webofscience.com/). Among the over 2000 articles found, we obtained the papers for the analysis of root trait response to drought based on the following criteria: 1) the experiment was conducted in well-controlled environments (e.g. pot, PVC tube, box, and small plots in field); 2) at least 3 biological replications were performed; and 3) data are available and obtainable under both well-watered and water-stressed conditions.

### Data extraction

Data for gene, QTL and hormones were extracted and summarized from the related papers. Data for the root trait analysis and 1-D distribution of root length density in the figures were extracted using GetData Graph Digitizer and data in the tables were copied.

### Data analysis

**Figure 2:**
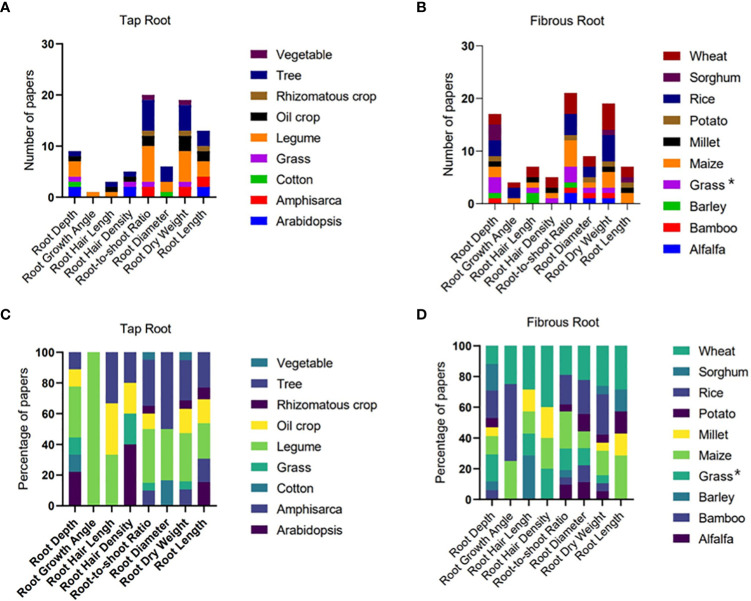
The number and percentage of papers reporting different root traits of different plant species with tap **(A, C)** and fibrous **(B, D)** root systems. Grasses in the tap root system include weed from Maganti et al (2005) and *Paspalum dilatatum* from Vasellati et al. (2001). Grasses in the fibrous root system (indicated by * in **(B, D)** include Perennial grass *Dactylis glomerata* from Bristiel et al. (2019), switchgrass from Liu et al. (2019), and perennial native grasses from Vega et al. (2020).

In the analysis of molecular manipulation of RSA under drought, we compiled a dataset of 109 records of genes and QTLs involved in the drought response of root traits in different plant species from 52 published papers; we also complied a dataset including 105 records of hormones from 29 published papers for the analysis. In the analysis of root traits in response to drought, we collected 808 pairs of data under well-watered and water-stressed conditions from 79 published papers. The number and percentage of papers reporting each trait in different species were presented (**Figure 2**). Ratio of drought response was calculated as the value of root traits observed under water-stressed condition divided by that under well-watered condition. Meta-analysis for drought response ratio of root traits was performed with the method from Zhou et al. (2018). In the study of one-dimensional root distribution to drought, 156 pairs of RLD data under well-watered and water-stressed conditions were collected from 6 published papers. Data were normalized with respect to the maximum values of sampling depth and RLD observed under each water availability condition in each selected paper. An exponential function was fitted between normalized depth and root length density. The number of 2-dimensional and 3-dimensional root distribution studies in response to drought was too small for a valid statistical analysis. Hence, we presented typical results in published studies as an example to discuss the effects of water deficit on 2-D root density distribution and 3-D RSA and its regulation mechanism.

## Results and discussion

### Genes, QTLs and hormones involved in the drought response of root traits

**Figure 3:**
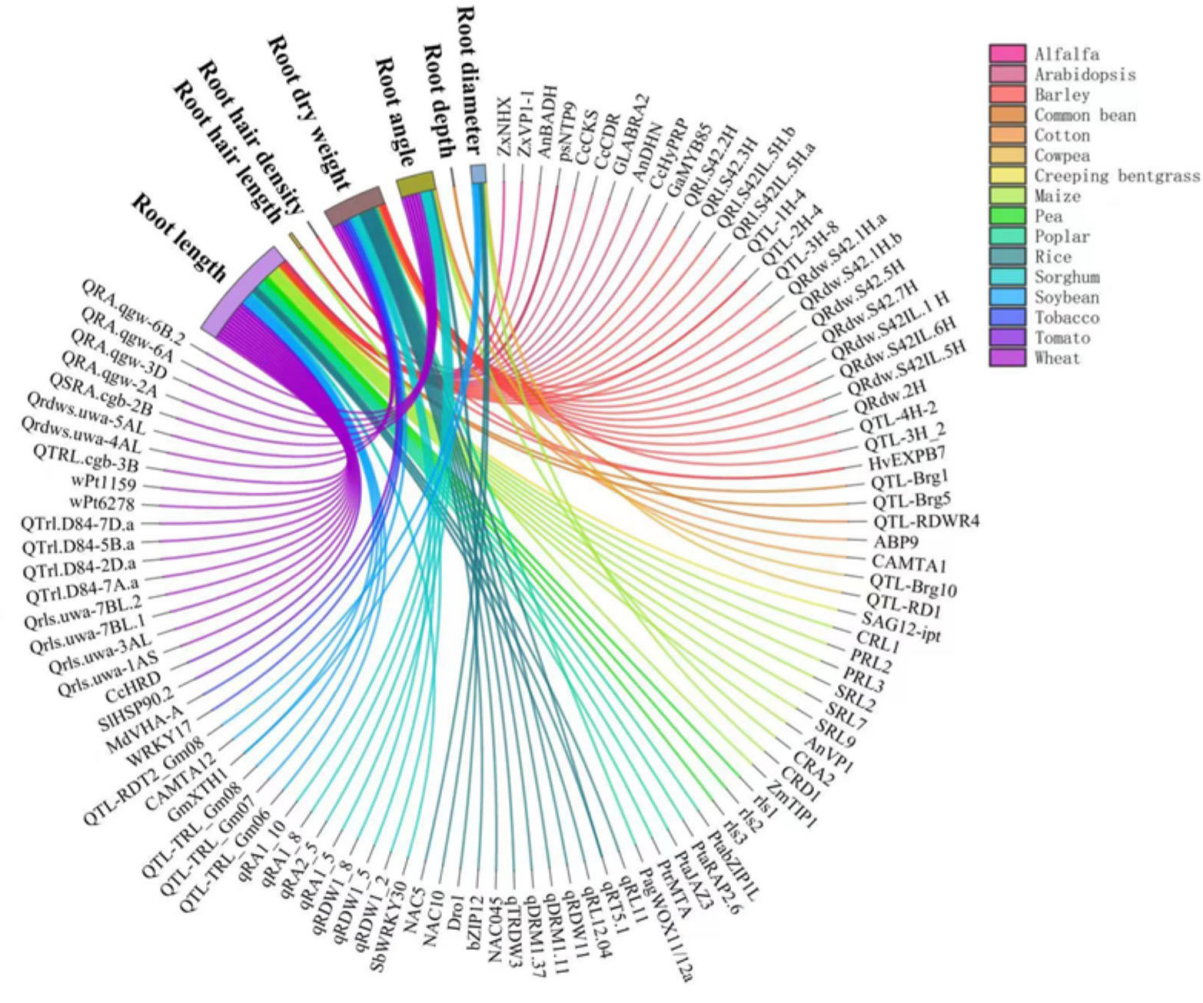
Genes and QTLs involved in the drought responses of root traits in different plant species reported in the literature. Different colors of lines and flows represent different crops. Data and references are included in **Supplementary Table 1** and **Supplementary File 1**, respectively.

The responses of RSA traits to drought are controlled by a complex regulation network involving sensing, signaling, and gene expression in a wide range of plants, as reviewed by Janiak et al. (2016). Several literature reviews have discussed these genes and QTLs in drought responses in cereal crops (Siddiqui et al., 2021), centering on rice (Kim et al., 2020), wheat (Kulkarni et al., 2017; Li et al., 2021), and grain legumes (Ye et al., 2018). A very recent review (Ranjan et al., 2022) has summarized the genes, QTLs, transcription factors, mRNAs involved in RSA responses to drought in a wide range of plants. A number of genes and QTLs governing the responses of RSA to drought have been identified in a variety of plants (**Figure 3**). Wheat is the most intensively studied crop, followed by rice and *Arabidopsis*. Among the root traits of interests, root length and root dry weight under drought are associated with the largest number of genes (**Figure 3**). These two traits reflect the overall growth of the root system and hence it has received sufficient attention. For instance, root length under drought was associated with QTLs including CRL1, PRL2, PRL3, SRL2, SRL7, and SRL9 in maize (Li et al., 2017) and QTRL.cgb-3B in wheat (Liu et al., 2013). Root dry weight under drought was associated with Qrdws.uwa-4AL and Qrdws.uwa-5AL in wheat (Ayalew et al., 2017) and qRDW1_2, qRDW1_5, and qRDW1_8 in sorghum (Mace et al., 2012). Root angle was regulated by DEEPER ROOTING 1 (*DRO1*), a rice quantitative trait locus and higher expression of DRO1 increases the root growth angle (Uga et al., 2013). Root diameter was enlarged by the overexpression of OsNAC5 (Jeong et al., 2013) and OsNAC10 (Jeong et al., 2010) in rice roots under drought. Very recently, the regulation of lateral root diameter by QHB and OsWOX10 has been identified under mild drought in rice (Kawai et al., 2022).

A recent genome-wide association study (GWAS) on the roots of two contrasting rice varieties under drought revealed 288 differentially expressed genes from the families of NAC, AP2/ERF, AUX/IAA, EXPANSIN, WRKY, and MYB (Abdirad et al., 2022). This finding warrants further research into verifying the roles of these genes in the differential RSA responses to water stress as one variety enhanced growth and root exploration to access water to avoid water deficit, whereas the other relied on cell insulation to maintain water and antioxidant system to withstand water stress (Abdirad et al., 2022).

We also covered species not yet been reviewed such as poplar (Zhou et al, 2020b), apple (Geng et al., 2018), and alfalfa (Wan et al., 2021) to complement published reviews (**Figure 3**). Poplar, a biofuel crop grown on marginal lands with insufficient water and nutrient resources, are the most well studied tree species in the molecular mechanism modifying RSA in response to drought (Dash et al, 2017; Dash et al, 2018; Wang et al., 2020). PtabZIP1-like gene was reported to enhance lateral root formation and biomass growth under drought stress (Dash et al., 2017). A recent study demonstrated that WUSCHEL-related homeobox gene PagWOX11/12a promoted root elongation and biomass growth in poplar in response to drought stress (Wang et al., 2020). Additionally, transgenic apple plants over expressing MdMYB88 or MdMYB124 had higher root-to-shoot ratios under long-term drought stress (Geng et al., 2018). Compared with annual crops, the genetic control of RSA in perennial trees to drought is poorly understood due to the lack effective phenotyping tools.

**Figure 4:**
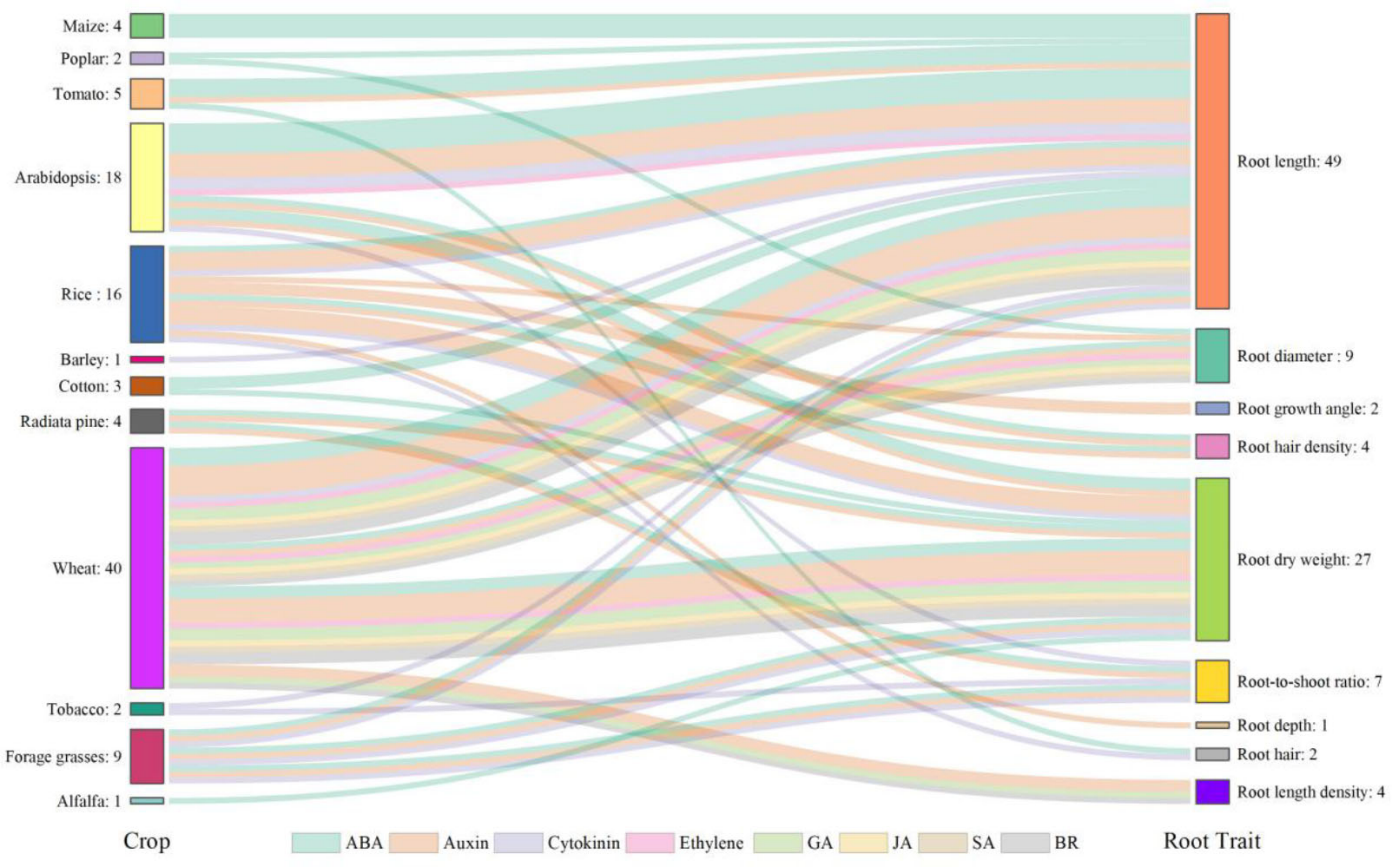
Hormones involved in the drought responses of root traits in different plant species reported in the literature. The numbers of observations are also shown. Crops are listed on the left-hand side and root traits on the right-hand side. Crops and root traits are connected by specific flows in different colors representing different hormones involved. Data and references are included in **Supplementary Table 2** and **Supplementary File 2**, respectively.

Plant hormones, such as abscisic acid (ABA), auxin, cytokinin, ethylene, gibberellic acid (GA), jasmonic acid (JA), salicylic acid (SA), and brassinosteroid (BR), are known to mediate root growth which contribute to development of RSA under normal and droughted conditions (Dalal et al., 2018; Karlova et al., 2021; Ranjan et al., 2022). Wheat, rice, and *Arabidopsis* are the most studied species (**Figure 4**) as they are the source for staple food for mankind or serve as the model plant in scientific research. Root length, root dry weight and root-to-shoot ratio are the most studied traits, as they are closely associated with the function of the root system (**Figure 4**). The number of studies involving ABA is the largest, followed by auxin and cytokinin (**Figure 4**). ABA plays the most critical role in regulating RSA. The records of ABA account for 34.3% of the total observations and it is involved in the regulation of almost all root traits under water stress (**Figure 4**). Moderate water stress in tomato increased the primary root length in the wild type but failed to enhance it in the mutant which lacked a fundamental gene in the ABA biosynthetic pathway and therefore had a lower ABA concentration compared to the wild type, suggesting that ABA played a positive role in mediating the regulation of primary root elongation under drought (Zhang et al., 2022). Higher expression of *DRO1* was shown to increase root growth angle under drought but it was negatively regulated by auxin (Uga et al., 2013). Cytokinin is known as a negative regulator of root growth (Ranjan et al., 2022) and the degradation of it was reported to increase the length of lateral roots and root dry weight, leading to improved drought tolerance in barley (Pospíšilová et al., 2016). In addition to a single hormone, Rowe et al. (2016) revealed how the hormonal network including ABA, auxin, ethylene, and cytokinin influence root growth under water stress. As the interplay of hormones in shaping RSA is very complex even under well-watered condition, drought will add a new layer of complexity and greater knowledge is needed to understand the complex hormonal crosstalk in the drought response of RSA.

ABA and auxin played roles in xerotropism, hydropatterning, and xerobrancing (Giehlvon Wiren, 2018; Orman-Ligeza et al., 2018; Dinneny, 2019; Lucob-Agustin et al., 2021), which contribute to the drought response of RSA. When water supply in soils is sufficient and homogeneous, the root system is likely to develop symmetrically around the root axis. When water availability in top soil layers is limited and sufficient water is retained in deep soil layers, auxin is involved in the mediation of root growth towards deep soil layers. This phenomenon is termed as xerotropism, in which the response of roots to gravity is enhanced to form deeper roots (Lucob-Agustin et al., 2021). When roots are exposed to differential water availabilities on either side of the root, formation of roots hairs and aerenchyma (plant tissues containing enlarged gas-filled intercellular spaces) is induced on the side exposed to air while formation of lateral roots are enhanced on the side in direct contact with water (Giehlvon Wiren, 2018). This phenomenon is termed as hydropatterning. Auxin has been reported to promote the initiation of lateral roots on the side in contact of water, determining whether and in which direction lateral roots form (Orosa-Puente et al., 2018). When roots encounter dry soil patches or air, ABA has been reported to repress lateral root formation there (xerobranching) (Orman-Ligeza et al., 2018). In soils that are not completely dry or flooded, an air-water interface forms between soil particles (Giehlvon Wiren, 2018). Such variation in soil water availability stimulates the growth towards water and this response, termed as hydrotropism, is also auxin-dependent (Lucob-Agustin et al., 2021). Hydrotropism guides the growth of roots to water while hydropatterning alters the distribution of root hairs and lateral roots along the circumference of the root surface (Giehlvon Wiren, 2018).

### Responses of typical root traits to drought

**Figure 5:**
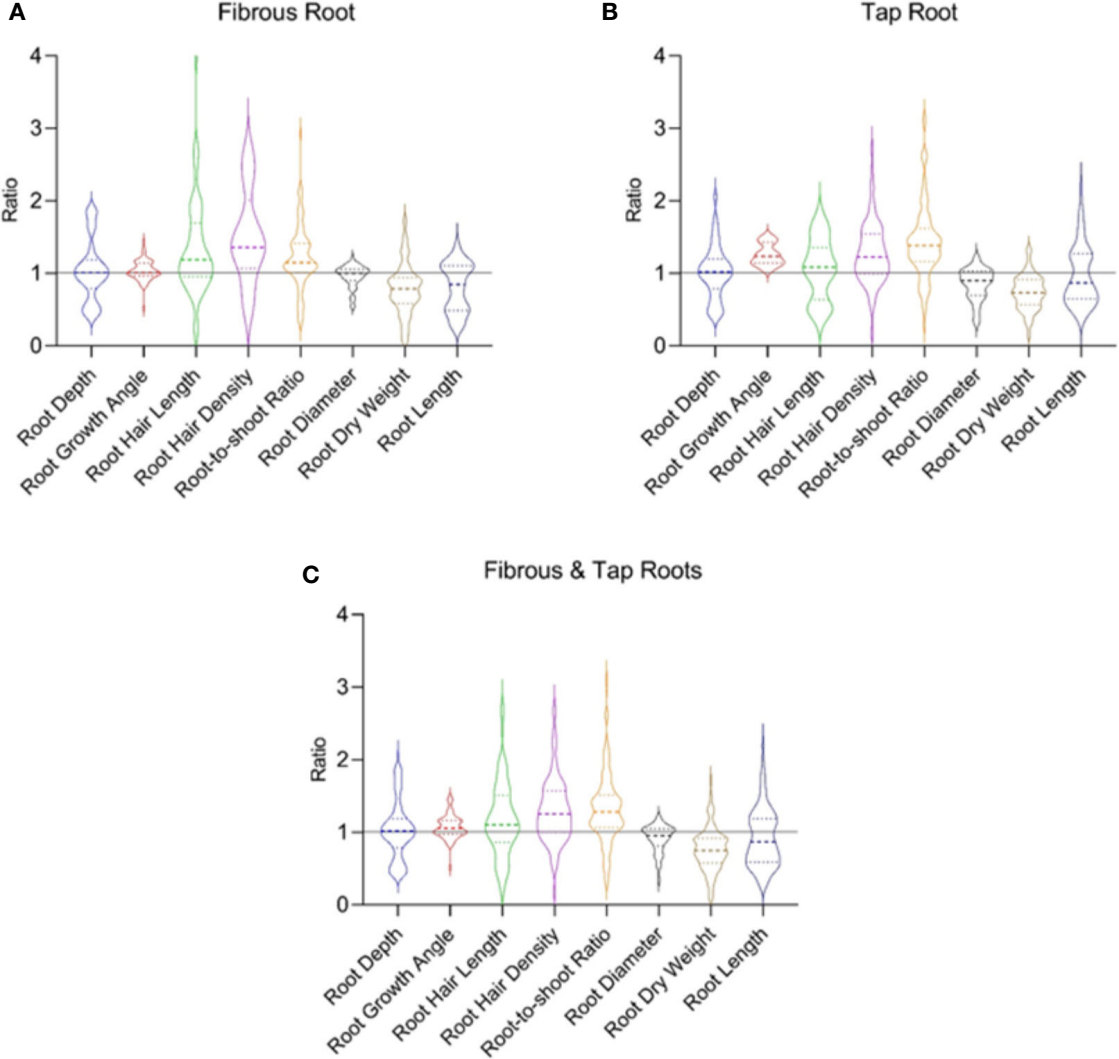
The drought response of typical root traits reported in the fibrous root **(A)** and tap root **(B)** systems in 70 published papers. **(C)** shows data from both the fibrous and tap root systems. Ratio is calculated as the value observed under water-stressed condition divided by that under well-watered condition. Data and references are included in **Supplementary Table 3** and **Supplementary File 3**, respectively.

Positive, negative, and null responses to drought in each root trait are reported in the literature and they are indicated by the ratio > 1, < 1, and =1, respectively (**Figure 5**). The inconsistent results are likely due to the different timing and intensity of water stress and the crops (Kato et al., 2006; Rauf and Sadaqat, 2007; Kamoshita et al., 2008; Kano et al., 2011; Padilla et al., 2013; Larson and Funk, 2016). The distribution of the ratio values determined the average ratio of each trait, which can be used to demonstrate the overall impact of drought on each trait (**Figure 5**). Due to the contrasting and diversified results (Olmo et al., 2014; Welles and Funk, 2021), generalizations need to be made very cautiously regarding the response of root traits to drought. The root traits in both the tap root and fibrous root systems follow a similar pattern in response to water stress (**Figure 5**).

In the fibrous root system (**Figure 5A**), water stress increased root hair density, root hair length and root-to-shoot ratio by 49.4%, 35.8%, and 21.6%, respectively. Water stress decreased root dry weight and root length by 21.9% and 19.8%, respectively. There are different reasons accounting for the increased root-to-shoot ratio in response to drought. In most cases, water stress decreased the dry matter accumulation in both shoot and root with a greater reduction in the root system, leading to an enhanced root-to-shoot ratio. Increased root dry weight accompanied by decreased shoot dry weight also occurred, which also caused increased root-to-shoot ratio. Although water stress had no effect on the mean of root depth, root growth angle, and root diameter, both positive and negative effects on the traits have been reported in the literature as reflected in the distribution of the ratio values (**Figure 5**). In the tap root system (**Figure 5B**), water stress caused an increase in root hair density, root growth angle, and root-to-shoot ratio by 27.3%, 25.3%, 42.6%, respectively. Water stress induced a decrease in root dry weight and root length by 26.5% and 3.4%, respectively. Similar to the fibrous root system, in most cases water stress decreased the dry weight in both shoot and root with a greater reduction in the root system, leading to an enhanced root-to-shoot ratio. Inconsistent responses to drought reported in each trait (**Figure 5**) reflected the different strategies plants used to deal with drought.

Root growth angle is generally defined as the degree between the horizontal and the root (Lynch, 2022). Larger root growth angles result in root elongation towards the deeper soil layers and this is believed to be an important trait for the access to and capture of deep soil water under drought (Vega et al., 2020; Lynch, 2022). Larger root growth angle contributed to higher yield as reported in maize (Ali et al., 2015) and rice (Uga et al., 2013) under water-limited conditions. Therefore, larger root growth angle is considered as a desirable trait of drought tolerance in the breeding program. However, some species do not increase root growth angle under drought (**Figure 5**). Introducing genes such as *DRO1* into the cultivar may enable the crop to avoid drought by increasing rooting depth and hence maintain yield under drought conditions. Growth angle largely determines root depth and larger rooting depth is associated with steeper root angles (An et al., 2017; Alahmad et al., 2019).

Root depth determines plants’ ability to capture water in the deep soil layers (Nippert and Holdo, 2015; Nardini et al., 2016). A deeper root system under drought is achieved by the continued and sustained growth of root systems even at extremely low water availabilities, which might increase the mechanical strength of the soil and penetration impedance for the roots (Rich and Watt, 2013). Root depth has been reported to affect seasonal progression of water status, gas exchange, and maintenance of vascular integrity (Nardini et al., 2016). Plants in different ecosystems differ considerably in rooting depth (Schenk and Jackson, 2002) and drought-tolerant species tended to be deep-rooted while drought-sensitive ones were shallow-rooted (Padilla and Pugnaire, 2007; Zhou et al., 2020a). Nevertheless, recent evidence in *Arabidopsis* has also shown that shallow rooting system is developed under drought for capturing water in the surface soil (Ogura et al., 2019). This new discovery challenges the conventional thinking of deep rooting and drought tolerance and opens up a debate over whether deep or shallow rooting benefits plants under drought. This shallow rooting pattern could occur in the ecosystem where rainfall happens within a short period of time. This shallow rooting strategy has also been adopted in succulent plants (e.g. cacti) in order to capture moisture in the topsoil owing to the light and brief desert rain (Schenk and Jackson, 2002). Nevertheless, it remains to be investigated whether this strategy is applicable to crops depending on the water availability in the soil profile. Water is mostly stored in the deep soil layer in the dryland agricultural system although water in the topsoil layer is temporarily available following sporadic rainfall. In contrast, water is constantly available in the topsoil layer during the growing season of the crops in the irrigated system.

Root hairs are specialized structures in the shape of tubular protrusions (typically 10 μm in diameter) arising from root epidermis and emerging behind the root elongation zone and they represent about 2% of the root mass (Marin et al., 2021). Root hair density and length are important traits affecting root water uptake (Mackay and Barber, 1987; Brown et al., 2012; Wang et al., 2016; Carminati et al., 2017). Negative and positive responses of these traits to water stress have been reported (**Figure 5**). Among the 22 wheat genotypes examined, most genotypes showed increased root hair density and length with only a couple of exceptions (Robin et al., 2021). Similar to the proliferation of lateral roots (Robin et al., 2021), increased root hairs significantly increase the contact area between roots and the surrounding soil (White and Kirkegaard, 2010) and facilitates water uptake under abiotic stresses (Xiao et al., 2020a; Xiao et al., 2020b; Kohli et al., 2022). Root hairs also play an important role in plant-microbe interaction (Fromm, 2019). Root hairs, together with mucilage secretion from roots and microbes, form the rhizosheath (Karanja et al., 2021; Zhang et al., 2021), which can facilitate the water uptake in the sheathed region under water deficit (Zhang et al., 2020). It remains to be investigated regarding the drought adaptive mechanism behind the genotypes without increased root hair proliferation. The possible adaptive strategies these plants adopted might include the changes in the root hydraulic properties (Leitner et al., 2014; Tron et al., 2015) or the osmotic adjustment of root cells (Palta and Turner, 2019), which also contributed to increased root water uptake.

Root diameter is a measure of root thickness. Building both finer and thicker roots in response to drought has been reported (**Figure 5**) and both responses are considered to benefit crops under drought. Building finer roots under drought is considered as a strategy for conserving resources when dry matter production is reduced (Henry et al., 2012). Finer roots may also benefit the crop under drought by increasing the surface to volume ratios of roots and hence the surface area of roots in contact with soil, allowing for increased withdrawal of water from the soil (Palta et al., 2011; Comas et al., 2013; Awad et al., 2018). On the other hand, thicker roots may be advantageous under drought because they have been hypothesized to be more capable of branching and producing more lateral roots, thereby increasing root length density and exploring deep soil layers and thus, enhancing drought tolerance (Ingram et al., 1994; Trillana et al., 2001). Rice plants that were genetically modified to have increased root diameter have been reported to have a higher yield under drought (Jeong et al., 2010; Jeong et al., 2013).

Root-to-shoot ratio is the ratio of the root and the shoot on a dry weight basis (Whitmore and Whalley, 2009). Increased root-to-shoot ratio under drought conditions has been widely reported and it is a drought avoidance strategy allowing the allocation of resource (dry matter) to the root for efficient water and nutrient acquisition (**Figure 5**). However, decreased root-to-shoot ratios have also been reported (**Figure 5**). Although continued investment in root growth reduced the risk of crop failure under water stress, it penalized shoot growth during dry periods. Particularly, continued root growth under drought could result in permanently retarded shoot growth at a critical developmental stage. Hence rather than grow a large root system in response to drought, a plant may allow part of the root system to die during drought and start new growth when favorable conditions return. These views proposed in much of the older literature were discussed in a review (Whitmore and Whalley, 2009). The responses of root-to-shoot ratio are also dependent on other factors such as cultivar (Ovalle et al., 2015; Moles et al., 2018) and the degree of drought (Liu and Li, 2005). Water restriction increased the root-to-shoot ratio of the deep-rooted saplings, while it had no significant effect on the shallow-rooted saplings (Ovalle et al., 2015). In an experiment where two spring wheat cultivars were subjected to mild and severe water stress, an increase in root-to-shoot ratio was observed under moderate water stress for the drought-tolerant cultivar and under severe water stress for the drought-sensitive cultivar (Liu and Li, 2005). This was explained by the significantly reduced respiration rate in the drought-tolerant cultivar under moderate drought. The diminished costs of maintaining root function allow the drought-tolerant plants to maintain a relatively large root system for water capture (Liu and Li, 2005).

Root length can be a measure of the overall growth of the root system. It is not surprising that inconsistent and dynamic responses of root length to drought have been reported (**Figure 5**). Total measurable root length did not necessarily reflect the root’s ability to take up water as it was argued that only a fraction of the total root length is active in water and nutrient uptake (Robinson et al., 1991; Sharma et al., 2018). Although the long and deep roots may not directly contribute to water uptake, they are functionally important in transporting the water and nutrients taken from active root parts to the rest of the plant (Sharma et al., 2018). Increased root length under drought condition may come at a significantly increased expense of root metabolic cost (Hasanuzzaman et al., 2019). Hence, for the genotypes that are capable of gaining more water by increasing root length at reduced metabolic costs, greater productivity can potentially be achieved under drought (Hasanuzzaman et al., 2019). Therefore, the selection of plant genotypes for long root length also needs to consider the metabolic cost to the plant, and otherwise increased metabolic cost could decrease the yield.

Root responses to drought are dependent on the soil types differing in texture, depth, water-holding capacity, and root penetration resistance (Cairns et al., 2004; Annicchiarico, 2007; Cairns et al., 2011; Menge et al., 2016; Palta and Turner, 2019). In the low rainfall environment (e.g. Mediterranean-type climate), clay soils are more susceptible to subsoil compaction and poor drainage than sandy soils (Palta and Turner, 2019), which may hinder the rapid profuse growth and proliferation of the root system. It was not surprising that faster early root growth improved grain yield and water use efficiency on deep sandy soils with low water-holding capacity while such advantage disappeared on clay soils with better water-holding capacity (Palta and Turner, 2019). Despite a decrease in root dry weight under drought in many studies (**Figure 5**), there was a trend towards the concentration of the roots in the topsoil layer (25–30 cm) in fine-textured soils (e.g. clay) under drought (Christian, 1977; Palta and Turner, 2019). This was attributed to the high soil water-holding ability, which might prevent most water from reaching deeper soil layers (Palta and Turner, 2019). For soils with a high root penetration resistance due to underground mechanical impedance, developing more lateral roots in the shallow soil layer in response to drought stress was an appropriate strategy; in contrast, for soils without or with a low penetration resistance, enhancing the root development into the deep wet soil layer under drought is more suitable (Menge et al., 2016). In agricultural practice, shallow soil is commonly seen in lowland fields containing a hardpan, which is approximately 20cm beneath the soil surface. Hardpan restricts root growth into deeper soil layers, hence water and nutrient uptake is limited in the shallow soil layer (Lucob-Agustin et al., 2021).

Root responses to drought are also associated with the life cycle of the plants (Thorup-Kristensen et al., 2020). Short-lived species might be more flexible and plastic to changes in soil water availability than species with a long life cycle. Compared with perennial plants, annual, short-lived plants may have a different strategy of capturing water in the soil (Lombardi et al., 2021). It was reported that in the semi-arid grasslands, short-lived grass species showed greater rhizosheath thickness and fine root development compared to successional climax grasses (Hartnett et al., 2013). These adaptive responses indicated that enhanced rhizosheath development, which aided in water uptake and retention the rhizosphere, and extensive fine root system, which aided in water acquisition, might be important traits of short-lived grasses coping with drought conditions. Many perennial species, such as trees, have a tap root system. It was shown that plasticity of root biomass allocation was lower in tap-rooted species than fibrous-rooted species (Fry et al., 2018). When water was only available in the deep soil layers under drought conditions, fibrous–rooted species changed their biomass allocation to evenly distribute their roots through the soil profile while tap-rooted species remained largely the same. The lack of response of tap-rooted species was attributed to the adaptation of taproots: they are already designed to forage in deeper soil layers and no change is necessary in response to drought (Fry et al., 2018).

### Responses of 1D/2D root length density distribution and 3D RSA to drought

**Figure 6:**
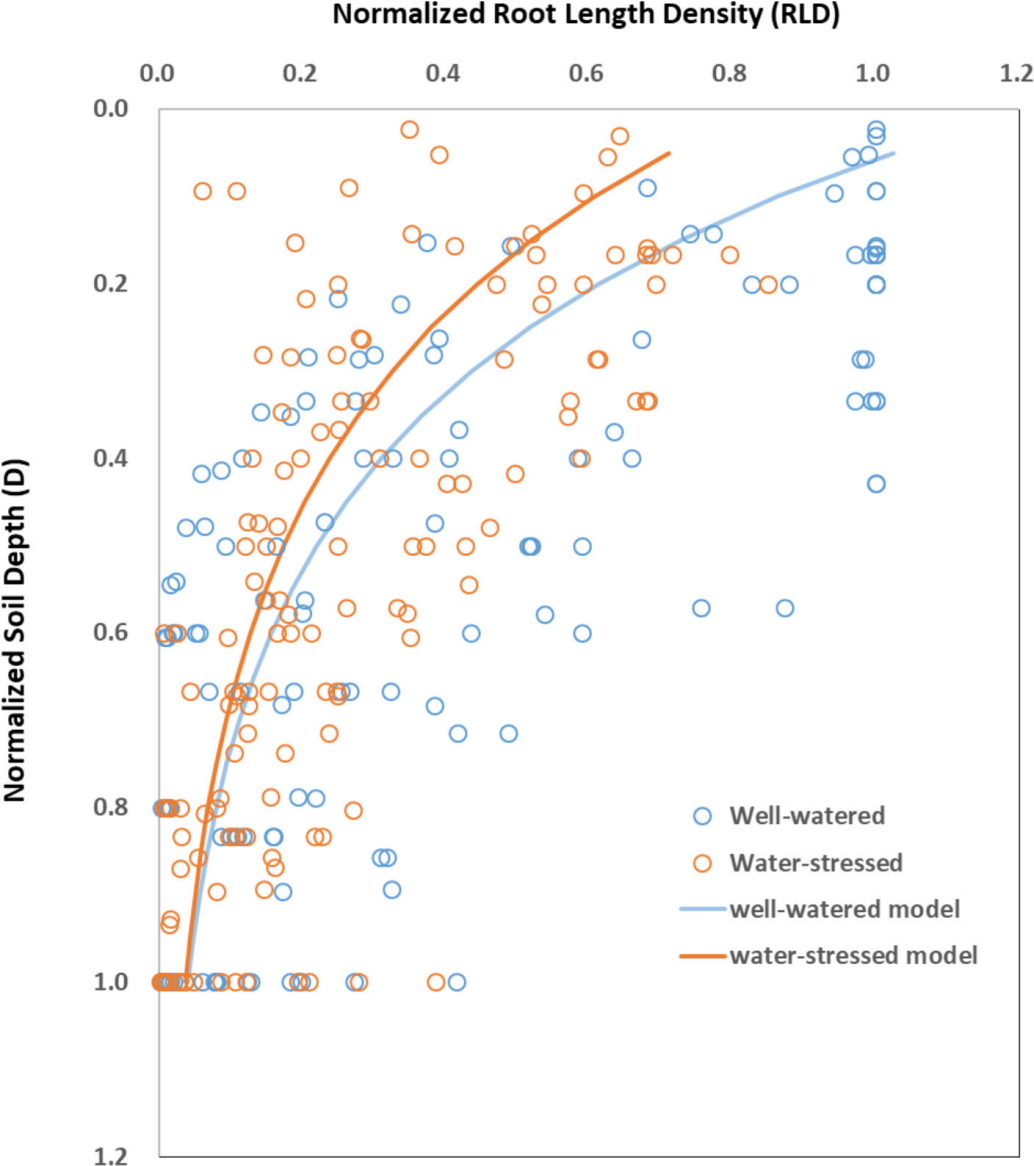
The distribution of root length density (RLD) at different soil depth (D) under well-watered (ww) and water-stressed (ws) conditions. Data are normalized with respect to the maximum values of soil depth and RLD observed under each water availability condition in each paper. Under well-watered condition, RLD_ww_=1.22×e^-3.42×D^ (n=154, R^2 =^ 0.41, P<0.001). Under water-stressed condition, RLD_ws_=0.83×e^-3.14×D^ (n=154, R^2 =^ 0.47, P<0.001). Original data are obtained from **Figure 4** in Zhan et al. (2015), **Figure 5** in Gao and Lynch (2016), **Figure 5** in Fitters et al. (2017), **Figure 2** in Ahmadi et al. (2018), **Figure 7** in Faye et al. (2019), and **Figure 5** in Hamedani et al. (2020).

Root length density (RLD) is generally described as the length of roots per unit of soil volume (Liang et al., 2017; El, Hassouni et al, 2018). RLD was larger in the surface soil layers and it decreased exponentially with soil depth (D) under both control and water stress conditions (**Figure 6**). Water stress decreased RLD almost at all depths and such decrease was most pronounced in the surface soil layers and it decreased with soil depth. The fitted equations between RLD and D areRLD_ww_=1.22×e^-3.42×D^ (n=154, R^2 =^ 0.41, P<0.001) for well-watered condition and RLD_ws_=0.83×e^-3.14×D^ (n=154, R^2 =^ 0.47, P<0.001) for water-stressed condition, respectively.

Similar to root dry weight and root length that reflect the growth of the whole root system (**Figure 4**), responses of RLD at the whole root level to drought are also inconsistent (Li et al., 2010; Padilla et al., 2015; Belachew et al., 2019). It has been argued that it is not the RLD of the whole root system, but the distribution of RLD in the soil profile is important for water extraction under drought (Vadez et al., 2013). A larger root system alone may not contribute much to drought tolerance if the large root portion is not distributed into moist soil (Jongrungklang et al., 2011). A number of studies have documented the distribution of RLD in response to drought in wheat (Zuo et al., 2006; Steinemann et al., 2015; Ali et al., 2018), barley (Ahmadi et al., 2020), chickpea (Purushothaman et al., 2017), sesame (Hamedani et al., 2020), maize (Zhan et al., 2015; Gao and Lynch, 2016; Jia et al., 2018), and melon (Sharma et al, 2018). These studies revealed different strategies in response to drought and in many cases RLD in the topsoil was more negatively affected by water stress (**Figure 6**). However, there are exceptions. RLD in the topsoil was increased in sorghum subjected to severe water deficit (Liang et al., 2017) and a similar increase was also reported in lettuce exposed to late drought (Kerbiriou et al., 2013). Although the interpretation for such increases is unclear, it might be a strategy to sustain shoot growth at the expense of root growth (Kerbiriou et al., 2013).

Increased RLD in deep soil layers under drought is generally considered as a worthy trait for plant breeders (Thangthong et al., 2018). The rice genotype with higher root length density in deep soil layers was reported to maintain dry matter production in two genotypes tested under a simulated rainfed lowland condition (Kameoka et al., 2015). The increase in drought duration makes this pattern more pronounced (Thangthong et al., 2016). However, the adjustment of RLD distribution under drought does not always confer yield and growth advantages depending on the genotype. A study demonstrated that both pearl millet lines (“SL28” and “LCICMB1”) reduced root growth in the dry topsoil layers and reoriented their root growth in deeper soil layers under drought conditions (Faye et al., 2019). However, SL28 showed a very strong and significant reduction in grain production in response to drought even though roots were reoriented to the deep soil layers. Similarly, it was reported that some peanut genotypes with high RLD under terminal drought had low yield (Koolachart et al., 2013). This inconsistent effect of RLD adjustment on crop yield suggested that RLD alone might not be used as a selection criterion for drought tolerance. It remains unclear how RLD distribution in conjunction with other root traits confer drought tolerance.

**Figure 7:**
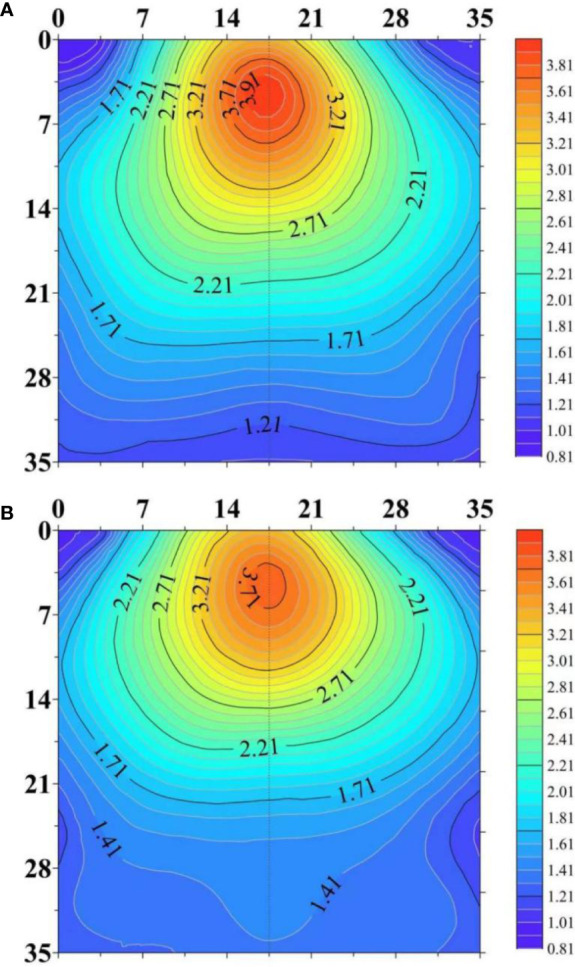
The horizontal and vertical distributions of root length density (RLD, cm cm^-3^) in the soil profile under well-watered **(A)** and water-stressed **(B)** conditions. The figure is adapted from **Figure 6** in Shabbir et al. (2021).

The center of RLD at 2-dimensional scale was shifted downwards in response to drought (**Figure 7**). RLD in the deep soil layer was increased under water stress while it was decreased in other layers of the soil. This change allows the roots to obtain water present in the deep soil layer. This change requires increased root growth angle and root depth (**Figure 4**). In agricultural practice, the response of 2-dimensioanl distribution of RLD to drought is affected by irrigation method (Oliveira et al., 1996; Perez-Pastor et al, 2014). The root system has been reported to tend to preferentially grow in the dripper zone under subsurface deficit irrigation. Perez-Pastor et al (2014) studied the RLD distribution of apricot trees in response to full irrigation at 100% of ETc (control), continuous deficit irrigation at 50% of ETc, and two regulated deficit irrigation. RLD values at the soil surface close to the drip-line band nearly doubled in the deficit irrigated treatments compared with those in the control treatment. In contrast, far from the drip line, RLD values in the control treatment were higher than those in all the deficit irrigation treatments. However, the tendency of growing towards emitters can adversely affect the utilization of water stored in the deeper soil profile. Hence, cultivars having the ability to extend root growth deeper in the soil profile under water deficit are promising to overcome the limitation of subsurface irrigation (Sharma et al., 2018).

The responses of horizontal and vertical distributions of RLD to water deficit were also dependent on whether the plant was grown in narrow deep soil or shallow wide soil (Zhang et al., 2019). Zhang et al. (2019) investigated the distribution of RLD of *Loliumperenne* L. (a perennial grass) grown in these two soil environments subjected to full irrigation and two levels of deficit irrigation. RLD tended to decrease from the center outward in the wide shallow soil, or from the surface to bottom in the narrow deep soil under all three treatments. The RLD in each soil ring in the shallow wide soil decreased gradually with decreased water supply, however, decreased supply had no significant effect on the distribution of RLD in the deep narrow soil.

Plant roots have diverse and complex 3D formations, which determines plants’ efficiency in acquiring water and nutrients in the soil (Morris et al, 2017). New technologies such as magnetic resonance imaging(MRI) and X-ray computer tomography (X-ray CT), ground penetrating radar (GPR) have been adopted to observe roots on 3D scale in the lab and the field (Rellan-Alvarez et al., 2015; Morris et al., 2017; Fan et al., 2022). 3D models have been developed to quantify RSA. Dunbabin et al. (2013) reviewed six widely used models including RootTyp, SimRoot, ROOTMAP, SPACSYS, R-SWMS, and RootBox. Until now, these models have aided in elucidating the root distribution and root function in interaction with variable environmental conditions (Leitner et al., 2010; Dunbabin et al., 2013) including drought. Here we present the key findings of 3D root distributions under drought. Using a combination of RootBoxand R-SWMS, Leitner et al. (2014) studied the role of root architectural and functional traits of maize in dealing with water stress at the flowering stage subjected to two hydrological scenarios (when most of the water is located at the top of the profile and when the water content increased in depth). The three phenotypes with different RLD profiles include P1 (with more roots at the top), P3 (with deeper rooting distribution), and P2 (an intermediate distribution). Simulations revealed that P1 was more efficient in water uptake than P2 and P3 when most of the water is located at the top, but has low ability to deal with a drier upper layer and that P3 outperformed P1 and P2 when the water content increased in depth. This corresponds to previous findings (e.g. Schenk and Jackson, 2002; Preti et al., 2010) showing that superiority of a given root architecture was dependent on the water regime. A deep and steep phenotype is beneficial only in situations where crop water supply depends on subsoil moisture while allocation of roots near the surface is more preferential when crops can rely on high in-season rainfall (Leitner et al., 2014). A similar conclusion was reached in a study modeling transpiration of 48 root architectures in 16 drought scenarios with distinct soil textures, rainfall distributions, and initial soil moisture availability (Tron et al., 2015). When sufficient rainfall is available before the growing season, root depth is a key trait for exploiting water stored in deep soil layers, especially in fine soils; when plant water supply mainly relies on rainfall events during the root system development, root density, especially near the soil surface, represents the most relevant trait for the exploration of soil moisture (Tron et al., 2015). These two studies (Leitner et al., 2014; Tron et al., 2015) also emphasized that that mere architectural description is insufficient to find root systems of optimum functionality and it required to consider the hydraulic traits of the root system, which did not fall into the scope of the current paper.

## Conclusion and prospect

Land plants adjust RSA to variable soil water availabilities for survival, development, and reproduction. Much progress has been made in the understanding of how the interplay of genes and hormones contribute to a single RSA trait (e.g. root growth angle) in different plants under drought (**Figures 3**–**5**). The inconsistent responses of a single RSA trait to drought (**Figure 5**) indicated that attention needs to be paid to understand how these RSA traits coordinate to shape the RSA at the 2- and 3- dimensional scales, which determines the function of the root system for optimal water uptake. Adjustment of RSA at the whole root system level in response to drought requires sensing the spatial and temporal dynamics of soil moisture and reallocating the dry matter within the root system at different soil depths. In the agricultural system, in order to breed optimal RSA suited for drought, it is important to exploit the mechanisms regulating root plasticity and sense ability at multiple levels. Other environmental stimuli and management strategies such as irrigation also impact RSA in the agricultural system. It is essential to gain an integrated multi-scale (molecular, cellular, tissue, and organ levels) understanding of the mechanisms regulating the drought responses of RSA. Such knowledge will aid in selecting and breeding crops with root ideotypes that are better adapted to different drought scenarios under different environments and management strategies. Given the complexity of RSA traits, high-throughput screening methods and GWAS will be helpful for in depth evaluating the drought adaptive responses of root system. A comprehensive and systematic understanding of RSA adaptive responses to drought at multiple levels will play an essential role in selection and breeding of drought tolerant crops, and water management in agricultural practice, which will help combat hunger and ensure food security in the context of global climate change.

## Data availability statement

The original contributions presented in the study are included in the article/**Supplementary Material.** Further inquiries can be directed to the corresponding author.

## Author contributions

JK conceived and designed the study. XK, WH, and JK made substantial contributions to the acquisition, analysis, and interpretation of the data. XK and WH wrote the draft of the article. JK and XK reviewed and corrected the manuscript. All authors approved the submitted manuscript and agreed to be listed.
