# Supplementary material for: Responses of root system architecture to water stress at multiple levels: A meta-analysis of trials under controlled conditions
Source: Front Plant Sci. 2022 Dec 9;13:1085409. doi: 10.3389/fpls.2022.1085409 (PMC9780461; doi:10.3389/fpls.2022.1085409)
Supplement: Supplementary file 1 [file Table_1.docx]

***Supplementary File1. Reference-Genes&QTLs***

Abdel-Ghani, A.H., Sharma, R., Wabila, C., Dhanagond, S., Owais, S.J., Duwayri, M.A., et al. (2019). Genome-wide association mapping in a diverse spring barley collection reveals the presence of QTL hotspots and candidate genes for root and shoot architecture traits at seedling stage. *BMC Plant Biol* 19, 216. doi:10.1186/s12870-019-1828-5

Arifuzzaman, M., Sayed, M.A., Muzammil, S., Pillen, K., Schumann, H., Naz, A.A., et al. (2014). Detection and validation of novel QTL for shoot and root traits in barley (*Hordeum vulgare* L.). *Mol. Breed.* 34, 1373-1387. doi:10.1007/s11032-014-0122-3

Ayalew, H., Liu, H., Börner, A., Kobiljski, B., Liu, C., and Yan, G. (2018). Genome-wide association mapping of major root length qtls under PEG induced water stress in wheat. *Front. Plant Sci.* 9, 1759. doi:10.3389/fpls.2018.01759

Ayalew, H., Liu, H., and Yan, G. (2017). Identification and validation of root length QTLs for water stress resistance in hexaploid wheat (*Titicum aestivum* L.). *Euphytica* 213, 126. doi:10.1007/s10681-017-1914-4

Bhattarai, U., and Subudhi, P.K. (2018). Identification of drought responsive QTLs during vegetative growth stage of rice using a saturated GBS-based SNP linkage map. *Euphytica* 214, 38. doi:10.1007/s10681-018-2117-3

Burridge, J.D., Schneider, H.M., Huynh, B.L., Roberts, P.A., Bucksch, A., and Lynch, J.P. (2017). Genome-wide association mapping and agronomic impact of cowpea root architecture. *Theor. Appl. Genet.* 130, 419-431. doi:10.1007/s00122-016-2823-y

Butt, H.I., Yang, Z., Gong, Q., Chen, E., Wang, X., Zhao, G., et al. (2017). *GaMYB85*, an R2R3 MYB gene, in transgenic *Arabidopsis* plays an important role in drought tolerance. *BMC Plant Biology* 17, 142. doi:10.1186/s12870-017-1078-3

Catolos, M., Sandhu, N., Dixit, S., Shamsudin, N.A.A., Naredo, M.E.B., Mcnally, K.L., et al. (2017). Genetic loci governing grain yield and root development under variable rice cultivation conditions. *Front. Plant Sci.* 8, 1763. doi:10.3389/fpls.2017.01763

Christopher, J., Christopher, M., Jennings, R., Jones, S., Fletcher, S., Borrell, A., et al. (2013). QTL for root angle and number in a population developed from bread wheats (*Triticum aestivum*) with contrasting adaptation to water-limited environments. *Theor. Appl. Genet.* 126, 1563-1574. doi:10.1007/s00122-013-2074-0

Dash, M., Yordanov, Y.S., Georgieva, T., Tschaplinski, T.J., Yordanova, E., and Busov, V. (2017). Poplar *PtabZIP1-like* enhances lateral root formation and biomass growth under drought stress. *Plant J.* 89, 692-705. doi:10.1111/tpj.13413

Dash, M., Yordanov, Y.S., Georgieva, T., Wei, H., and Busov, V. (2018). Gene network analysis of poplar root transcriptome in response to drought stress identifies a *PtaJAZ3PtaRAP2.6*-centered hierarchical network. *PLoS One* 13, e0208560. doi:10.1371/journal.pone.0208560

Dong, Q.L., Wang, C.R., Liu, D.D., Hu, D.G., Fang, M.J., You, C.X., et al. (2013). *MdVHA-A* encodes an apple subunit A of vacuolar H^+^-ATPase and enhances drought tolerance in transgenic tobacco seedlings. *J. Plant Physiol.* 170, 601-609. doi:https://doi.org/10.1016/j.jplph.2012.12.014

Dossa, K., Zhou, R., Li, D., Liu, A., Qin, L., Mmadi, M.A., et al. (2021). A novel motif in the 5'-UTR of an orphan gene 'Big Root Biomass' modulates root biomass in sesame. *Plant Biotechnol. J.* 19, 1065-1079. doi:10.1111/pbi.13531

Fondevilla, S., Fernández-Aparicio, M., Satovic, Z., Emeran, A.A., Torres, A.M., Moreno, M.T., et al. (2010). Identification of quantitative trait loci for specific mechanisms of resistance to *Orobanche crenata* Forsk. in pea (*Pisum sativum* L.). *Mol. Breed.* 25, 259-272. doi:10.1007/s11032-009-9330-7

Geng, D., Chen, P., Shen, X., Zhang, Y., Li, X., Jiang, L., et al. (2018). *MdMYB88* and *MdMYB124* enhance drought tolerance by modulating root vessels and cell walls in apple. *Plant Physiol.* 178, 1296-1309. doi:10.1104/pp.18.00502

Guo, X., Zhang, L., Zhu, J., Wang, A., and Liu, H. (2017). Christolea crassifolia *HARDY* gene enhances drought stress tolerance in transgenic tomato plants. *Plant Cell, Tissue and Organ Culture (PCTOC)* 129, 469-481. doi:10.1007/s11240-017-1192-9

Han, J.H., Shin, N.H., Jang, S., Yu, Y., Chin, J.H., and Yoo, S.C. (2018). Identification of quantitative trait loci for vigorous root development under water-deficiency conditions in rice. *Plant Breed. and Biotech.* 6. doi:10.9787/PBB.2018.6.2.147

He, X., Zeng, J., Cao, F., Ahmed, I.M., Zhang, G., Vincze, E., et al. (2015). *HvEXPB7*, a novel β-expansin gene revealed by the root hair transcriptome of Tibetan wild barley, improves root hair growth under drought stress. *J. Exp. Bot.* 66, 7405-7419. doi:10.1093/jxb/erv436

Hu, H., Dai, M., Yao, J., Xiao, B., Li, X., Zhang, Q., et al. (2006). Overexpressing a NAM, ATAF, and CUC (NAC) transcription factor enhances drought resistance and salt tolerance in rice. *Proc. Natl. Acad. Sci. U.S.A.* 103, 12987-12992. doi:10.1073/pnas.0604882103

Ibrahim, S., Schubert, A., Pillen, K., and Léon, J. (2012). QTL analysis of drought tolerance for seedling root morphological traits in an advanced backcross population of spring wheat. *Int. J. AgriSci.* 2, 619-629.

Jeong, J.S., Kim, Y.S., Baek, K.H., Jung, H., Ha, S.-H., Do Choi, Y., et al. (2010). Root-specific expression of O*sNAC10* improves drought tolerance and grain yield in rice under field drought conditions. *Plant Physiol.* 153, 185-197. doi:10.1104/pp.110.154773

Jeong, J.S., Kim, Y.S., Redillas, M.C.F.R., Jang, G., Jung, H., Bang, S.W., et al. (2013). *OsNAC5* overexpression enlarges root diameter in rice plants leading to enhanced drought tolerance and increased grain yield in the field. *Plant Biotechnol. J.* 11, 101-114. doi:https://doi.org/10.1111/pbi.12011

Joo, J., Lee, Y.H., and Song, S.I. (2014). Overexpression of the rice basic leucine zipper transcription factor *OsbZIP12* confers drought tolerance to rice and makes seedlings hypersensitive to ABA. *Plant Biotechnol. Rep.* 8, 431-441. doi:10.1007/s11816-014-0335-2

Kang, P., Bao, A.K., Kumar, T., Pan, Y.Q., Bao, Z., Wang, F., et al. (2016). Assessment of stress tolerance, productivity, and forage quality in T(1) transgenic alfalfa co-overexpressing *ZxNHX* and *ZxVP1-1* from zygophyllum xanthoxylum. *Front. Plant Sci.* 7, 1598. doi:10.3389/fpls.2016.01598

Li, P., Zhang, Y., Yin, S., Zhu, P., Pan, T., Xu, Y., et al. (2018). QTL-by-environment interaction in the response of maize root and shoot traits to different water regimes. *Front. Plant Sci.* 9. doi:10.3389/fpls.2018.00229

Liao, H., Yan, X., Rubio, G., Beebe, S.E., Blair, M.W., and Lynch, J.P. (2004). Genetic mapping of basal root gravitropism and phosphorus acquisition efficiency in common bean. *Funct. Plant Biol.* 31, 959-970. doi:10.1071/fp03255

Liu, X., Li, R., Chang, X., and Jing, R. (2013). Mapping QTLs for seedling root traits in a doubled haploid wheat population under different water regimes. *Euphytica* 189, 51-66. doi:10.1007/s10681-012-0690-4

Lo, T.S., Le, H.D., Nguyen, V.T., Chu, H.H., Le, V.-S., and Chu, H.M. (2015). Overexpression of a soybean expansin gene, *GmEXP1*, improvesdrought tolerance in transgenic tobacco. *Turk. J. of Bot.* 39, 988-995. doi:10.3906/bot-1502-40

Lu, L., Zhang, Y., He, Q., Qi, Z., Zhang, G., Xu, W., et al. (2020). MTA, an RNA m(6)A methyltransferase, enhances drought tolerance by regulating the development of trichomes and roots in poplar. *Int. J. Mol. Sci.* 21. doi:10.3390/ijms21072462

Mace, E.S., Singh, V., Van Oosterom, E.J., Hammer, G.L., Hunt, C.H., and Jordan, D.R. (2012). QTL for nodal root angle in sorghum (*Sorghum bicolor* L. Moench) co-locate with QTL for traits associated with drought adaptation. *Theor. Appl. Genet.* 124, 97-109. doi:10.1007/s00122-011-1690-9

Marowa, P., Ding, A., Xu, Z., and Kong, Y. (2020). Overexpression of *NtEXPA11* modulates plant growth and development and enhances stress tolerance in tobacco. *Plant Physiol. Biochem.* 151, 477-485. doi:10.1016/j.plaphy.2020.03.033

Naz, A.A., Arifuzzaman, M., Muzammil, S., Pillen, K., and Léon, J. (2014). Wild barley introgression lines revealed novel QTL alleles for root and related shoot traits in the cultivated barley (*Hordeum vulgare* L.). *BMC Genet.* 15, 107. doi:10.1186/s12863-014-0107-6

Noman, M., Jameel, A., Qiang, W.D., Ahmad, N., Liu, W.C., Wang, F.W., et al. (2019). Overexpression of *GmCAMTA12* enhanced drought tolerance in *Arabidopsis* and soybean. *Int. J. Mol. Sci.* 20. doi:10.3390/ijms20194849

Oyiga, B.C., Palczak, J., Wojciechowski, T., Lynch, J.P., Naz, A.A., Léon, J., et al. (2020). Genetic components of root architecture and anatomy adjustments to water-deficit stress in spring barley. *Plant Cell Environ.* 43, 692-711. doi:10.1111/pce.13683

Pandey, N., Ranjan, A., Pant, P., Tripathi, R.K., Ateek, F., Pandey, H.P., et al. (2013). *CAMTA 1* regulates drought responses in *Arabidopsis* thaliana. *BMC Genom.* 14, 216. doi:10.1186/1471-2164-14-216

Prince, S.J., Song, L., Qiu, D., Maldonado Dos Santos, J.V., Chai, C., Joshi, T., et al. (2015). Genetic variants in root architecture-related genes in a *Glycine soja* accession, a potential resource to improve cultivated soybean. *BMC Genom.* 16, 132. doi:10.1186/s12864-015-1334-6

Priyanka, B., Sekhar, K., Reddy, V.D., and Rao, K.V. (2010). Expression of pigeonpea hybrid-proline-rich protein encoding gene (*CcHyPRP*) in yeast and *Arabidopsis* affords multiple abiotic stress tolerance. *Plant Biotechnol. J.* 8, 76-87. doi:10.1111/j.1467-7652.2009.00467.x

Rahman, H., Pekic, S., Lazic-Jancic, V., Quarrie, S.A., Shah, S.M., Pervez, A., et al. (2011). Molecular mapping of quantitative trait loci for drought tolerance in maize plants. *Genet. Mol. Res.* 10, 889-901. doi:10.4238/vol10-2gmr1139

Reinert, S., Kortz, A., Léon, J., and Naz, A.A. (2016). Genome-wide association mapping in the global diversity set reveals new QTL controlling root system and related shoot variation in barley. *Front. Plant Sci.* 7, 1061. doi:10.3389/fpls.2016.01061

Sabar, M., Shabir, G., Shah, S.M., Aslam, K., Naveed, S.A., and Arif, M. (2019). Identification and mapping of QTLs associated with drought tolerance traits in rice by a cross between Super Basmati and IR55419-04. *Breed. Sci.* 69, 169-178. doi:10.1270/jsbbs.18068

Sun, X., Luo, X., Sun, M., Chen, C., Ding, X., Wang, X., et al. (2014). A *Glycine soja* 14-3-3 protein GsGF14o participates in stomatal and root hair development and drought tolerance in *Arabidopsis* thaliana. *Plant Cell Physiol.* 55, 99-118. doi:10.1093/pcp/pct161

Sun, Y., Liu, L., Sun, S., Han, W., Irfan, M., Zhang, X., et al. (2021). *AnDHN*, a dehydrin protein from ammopiptanthus nanus, mitigates the negative effects of drought stress in plants. *Front. Plant Sci.* 12, 788938. doi:10.3389/fpls.2021.788938

Tamirisa, S., Reddy, V.D., and Rao, K.V. (2014). Ectopic expression of pigeonpea cold and drought regulatory protein (CcCDR) in yeast and tobacco affords multiple abiotic stress tolerance. *Plant Cell, Tissue and Organ Culture (PCTOC)* 119, 489-499. doi:10.1007/s11240-014-0549-6

Tamirisa, S., Vudem, D.R., and Khareedu, V.R. (2017). A cyclin dependent kinase regulatory subunit (CKS) gene of pigeonpea imparts abiotic stress tolerance and regulates plant growth and development in *Arabidopsis*. *Front. Plant Sci.* 8, 165. doi:10.3389/fpls.2017.00165

Uga, Y., Okuno, K., and Yano, M. (2011). *Dro1*, a major QTL involved in deep rooting of rice under upland field conditions. *J. Exp. Bot.* 62, 2485-2494. doi:10.1093/jxb/erq429

Uga, Y., Sugimoto, K., Ogawa, S., Rane, J., Ishitani, M., Hara, N., et al. (2013). Control of root system architecture by *DEEPER ROOTING* *1* increases rice yield under drought conditions. *Nat. Genet.* 45, 1097-1102. doi:10.1038/ng.2725

Veerappa, R., Slocum, R.D., Siegenthaler, A., Wang, J., Clark, G., and Roux, S.J. (2019). Ectopic expression of a pea apyrase enhances root system architecture and drought survival in *Arabidopsis* and soybean. *Plant Cell Environ.* 42, 337-353. doi:10.1111/pce.13425

Wang, L.Q., Li, Z., Wen, S.S., Wang, J.N., Zhao, S.T., and Lu, M.Z. (2020a). WUSCHEL-related homeobox gene *PagWOX11/12a* responds to drought stress by enhancing root elongation and biomass growth in poplar. *J. Exp. Bot.* 71, 1503-1513. doi:10.1093/jxb/erz490

Wang, N.N., Xu, S.W., Sun, Y.L., Liu, D., Zhou, L., Li, Y., et al. (2019). The cotton WRKY transcription factor (*GhWRKY33*) reduces transgenic *Arabidopsis* resistance to drought stress. *Sci. Rep.* 9, 724. doi:10.1038/s41598-018-37035-2

Wang, X., Bi, S., Wang, L., Li, H., Gao, B.A., Huang, S., et al. (2020b). *GLABRA2* regulates actin bundling protein VILLIN1 in root hair growth in response to osmotic stress. *Plant Physiol.* 184, 176-193. doi:10.1104/pp.20.00480

Xiu, Y., Iqbal, A., Zhu, C., Wu, G., Chang, Y., Li, N., et al. (2016). Improvement and transcriptome analysis of root architecture by overexpression of Fraxinus pennsylvanica *DREB2A* transcription factor in *Robinia pseudoacacia* L. 'Idaho'. *Plant Biotechnol. J.* 14, 1456-1469. doi:10.1111/pbi.12509

Xu, Y., Burgess, P., Zhang, X., and Huang, B. (2016). Enhancing cytokinin synthesis by overexpressing ipt alleviated drought inhibition of root growth through activating ROS-scavenging systems in *Agrostis stolonifera*. *J. Exp. Bot.* 67, 1979-1992. doi:10.1093/jxb/erw019

Yadav, P.K., Gupta, N., Verma, V., and Gupta, A.K. (2021). Overexpression of *SlHSP90.2* leads to altered root biomass and architecture in tomato (*Solanum lycopersicum*). *Physiol. Mol. Biol. Plants* 27, 713-725. doi:10.1007/s12298-021-00976-6

Yan, H., Jia, H., Chen, X., Hao, L., An, H., and Guo, X. (2014). The cotton WRKY transcription factor *GhWRKY17* functions in drought and salt stress in transgenic Nicotiana benthamiana through ABA signaling and the modulation of reactive oxygen species production. *Plant Cell Physiol.* 55, 2060-2076. doi:10.1093/pcp/pcu133

Yang, Z., Chi, X., Guo, F., Jin, X., Luo, H., Hawar, A., et al. (2020). *SbWRKY30* enhances the drought tolerance of plants and regulates a drought stress-responsive gene, *SbRD19*, in sorghum. *J. Plant Physiol.* 246-247, 153142. doi:10.1016/j.jplph.2020.153142

Yu, H.Q., Zhou, X.Y., Wang, Y.-G., Zhou, S.F., Fu, F.L., and Li, W.C. (2017). A betaine aldehyde dehydrogenase gene from Ammopiptanthus nanus enhances tolerance of *Arabidopsis* to high salt and drought stresses. *Plant Growth Regul.* 83, 265-276. doi:10.1007/s10725-016-0245-0

Yu, H., Qu, J., Guo, X., Li, L., Zhang, X., Yang, Q., et al. (2022). Overexpression of vacuolar H^+^-pyrophosphatase (H^+^-PPase) gene from Ammopiptanthus nanus enhances drought tolerance in maize. *J. Agron. Crop Sci.* 208, 633-644. doi:10.1111/jac.12504

Zhang, X., Mi, Y., Mao, H., Liu, S., Chen, L., and Qin, F. (2020). Genetic variation in *ZmTIP1* contributes to root hair elongation and drought tolerance in maize. *Plant Biotechnol. J.* 18, 1271-1283. doi:10.1111/pbi.13290

Zhang, Y., Zhang, H.Z., Fu, J.Y., Du, Y.Y., Qu, J., Song, Y., et al. (2021). The *GmXTH1* gene improves drought stress resistance of soybean seedlings. *Mol. Breed.* 42, 3. doi:10.1007/s11032-021-01258-5
