# Supplementary material for: Responses of root system architecture to water stress at multiple levels: A meta-analysis of trials under controlled conditions
Source: Front Plant Sci. 2022 Dec 9;13:1085409. doi: 10.3389/fpls.2022.1085409 (PMC9780461; doi:10.3389/fpls.2022.1085409)
Supplement: Supplementary file 2 [file Table_2.docx]

***Supplementary File2. Reference-Hormones***

Chen, G., Liu, C., Gao, Z., Zhang, Y., Zhu, L., Hu, J., et al. (2018). Driving the expression of *RAA1* with a drought-responsive promoter enhances root growth in rice, its accumulation of potassium and its tolerance to moisture stress. *Environ. Exp. Bot.* 147, 147-156. doi:10.1016/j.envexpbot.2017.12.008

Cheng, S., Zhou, D.X., and Zhao, Y. (2016). WUSCHEL-related homeobox gene *WOX11* increases rice drought resistance by controlling root hair formation and root system development. *Plant Signal. Behav.* 11, e1130198. doi:10.1080/15592324.2015.1130198

Dalal, M., Sahu, S., Tiwari, S., Rao, A.R., and Gaikwad, K. (2018). Transcriptome analysis reveals interplay between hormones, ROS metabolism and cell wall biosynthesis for drought-induced root growth in wheat. *Plant Physiol. Biochem.* 130, 482-492. doi:10.1016/j.plaphy.2018.07.035

De Diego, N., Rodríguez, J.L., Dodd, I.C., Pérez-Alfocea, F., Moncaleán, P., and Lacuesta, M. (2013). Immunolocalization of IAA and ABA in roots and needles of radiata pine (*Pinus radiata*) during drought and rewatering. *Tree Physiol.* 33, 537-549. doi:10.1093/treephys/tpt033

Gulzar, F., Fu, J., Zhu, C., Yan, J., Li, X., Meraj, T.A., Shen, Q., Hassan, B., and Wang, Q. (2021). Maize WRKY transcription factor *ZmWRKY79* positively regulates drought tolerance through elevating ABA biosynthesis. *Int. J. Mol. Sci.* 22. doi:10.3390/ijms221810080

Han, H., Tian, Z., Fan, Y., Cui, Y., Cai, J., Jiang, D., et al. (2015). Water-deficit treatment followed by re-watering stimulates seminal root growth associated with hormone balance and photosynthesis in wheat (*Triticum aestivum* L.) seedlings. *Plant Growth Regul.* 77, 201-210. doi:10.1007/s10725-015-0053-y

Jeong, J.S., Kim, Y.S., Redillas, M.C.F.R., Jang, G., Jung, H., Bang, S.W., et al. (2013). *OsNAC5* overexpression enlarges root diameter in rice plants leading to enhanced drought tolerance and increased grain yield in the field. *Plant Biotechnol. J.* 11 1, 101-114. doi:10.1111/pbi.12011

Karanja, J.K., Aslam, M.M., Qian, Z., Yankey, R., Dodd, I.C., and Weifeng, X. (2021). Abscisic acid mediates drought-enhanced rhizosheath formation in tomato. *Front. Plant. Sci.* 12, 658787. doi:10.3389/fpls.2021.658787

Lee, M., Jung, J.H., Han, D.Y., Seo, P.J., Park, W.J., and Park, C.M. (2012). Activation of a flavin monooxygenase gene YUCCA7 enhances drought resistance in *Arabidopsis*. *Planta* 235, 923-938. doi:10.1007/s00425-011-1552-3

Li, S., Nie, Z., Sun, J., Li, X., and Yang, G. (2022). The physiological role of abscisic acid in regulating root system architecture of alfalfa in its adaptation to water deficit. *Agronomy* 12, 1882. doi:10.3390/agronomy12081882

Li, Z., Liu, C., Zhang, Y., Wang, B., Ran, Q., and Zhang, J. (2019). The bHLH family member *ZmPTF1* regulates drought tolerance in maize by promoting root development and abscisic acid synthesis. *J. Exp. Bot.* 70, 5471-5486. doi:10.1093/jxb/erz307

Liang, C., Meng, Z., Meng, Z., Malik, W., Yan, R., Lwin, K.M., et al. (2016). *GhABF2*, a bZIP transcription factor, confers drought and salinity tolerance in cotton (*Gossypium hirsutum* L.). *Sci Rep* 6, 35040. doi:10.1038/srep35040

Ma, H., Liu, C., Li, Z., Ran, Q., Xie, G., Wang, B., et al. (2018). *ZmbZIP4* contributes to stress resistance in maize by regulating ABA synthesis and root development. *Plant Physiol.* 178, 753-770. doi:10.1104/pp.18.00436

Mao, H., Li, S., Wang, Z., Cheng, X., Li, F., Mei, F., et al. (2020). Regulatory changes in *TaSNAC8-6A* are associated with drought tolerance in wheat seedlings. *Plant Biotechnol. J.* 18, 1078-1092. doi:10.1111/pbi.13277

Perlikowski, D., Augustyniak, A., Masajada, K., Skirycz, A., Soja, A.M., Michaelis, Ä., et al. (2019). Structural and metabolic alterations in root systems under limited water conditions in forage grasses of Lolium-Festuca complex. *Plant Sci.* 283, 211-223. doi:10.1016/j.plantsci.2019.02.001

Placido, D.F., Campbell, M.T., Folsom, J.J., Cui, X., Kruger, G.R., Baenziger, P.S., et al. (2013). Introgression of novel traits from a wild wheat relative improves drought adaptation in wheat. *Plant Physiol.* 161, 1806-1819. doi:10.1104/pp.113.214262

Placido, D.F., Sandhu, J., Sato, S.J., Nersesian, N., Quach, T., Clemente, T.E., et al. (2020). The *LATERAL ROOT DENSITY* gene regulates root growth during water stress in wheat. *Plant Biotechnol. J.* 18, 1955-1968. doi:10.1111/pbi.13355

Pospíšilová, H., Jiskrová, E., Vojta, P., Mrízová, K., Kokáš, F., Čudejková, M.M., et al. (2016). Transgenic barley overexpressing a cytokinin dehydrogenase gene shows greater tolerance to drought stress. *N. Biotechnol.* 33, 692-705. doi:10.1016/j.nbt.2015.12.005

Rosales, M.A., Maurel, C., and Nacry, P. (2019). Abscisic acid coordinates dose-dependent developmental and hydraulic responses of roots to water deficit. *Plant Physiol.* 180, 2198-2211. doi:10.1104/pp.18.01546

Rowe, J.H., Topping, J.F., Liu, J., and Lindsey, K. (2016). Abscisic acid regulates root growth under osmotic stress conditions via an interacting hormonal network with cytokinin, ethylene and auxin. *New phytol.* 211, 225-239. doi:10.1111/nph.13882

Seo, P.J., Xiang, F., Qiao, M., Park, J.Y., Lee, Y.N., Kim, S.G., et al. (2009). The *MYB96* transcription factor mediates abscisic acid signaling during drought stress response in *Arabidopsis*. *Plant Physiol.* 151, 275-289. doi:10.1104/pp.109.144220

Sharp, R.E., Wu, Y., Voetberg, G., Saab, I.N., and Lenoble, M.E. (1994). Confirmation that abscisic acid accumulation is required for maize primary root elongation at low water potentials. *J. Exp. Bot.* 45, 1743-1751. doi:10.1093/jxb/45.Special_Issue.1743

Uga, Y., Sugimoto, K., Ogawa, S., Rane, J., Ishitani, M., Hara, N., et al. (2013). Control of root system architecture by *DEEPER ROOTING* *1* increases rice yield under drought conditions. *Nat. Genet.* 45, 1097-1102. doi:10.1038/ng.2725

Werner, T., Nehnevajova, E., Köllmer, I., Novák, O., Strnad, M., Krämer, U., et al. (2010). Root-specific reduction of cytokinin causes enhanced root growth, drought tolerance, and leaf mineral enrichment in *Arabidopsis* and tobacco. *Plant Cell* 22, 3905-3920. doi:10.1105/tpc.109.072694

Xu, W., Jia, L., Shi, W., Liang, J., Zhou, F., Li, Q., et al. (2013). Abscisic acid accumulation modulates auxin transport in the root tip to enhance proton secretion for maintaining root growth under moderate water stress. *New Phytol.* 197, 139-150. doi:10.1111/nph.12004

Yu, L.H., Wu, S.J., Peng, Y.S., Liu, R.N., Chen, X., Zhao, P., et al. (2016). *Arabidopsis* EDT1/HDG11 improves drought and salt tolerance in cotton and poplar and increases cotton yield in the field. *Plant Biotechnol. J.* 14, 72-84. doi:10.1111/pbi.12358

Zhang, Q., Yuan, W., Wang, Q., Cao, Y., Xu, F., Dodd, I.C., et al. (2022). ABA regulation of root growth during soil drying and recovery can involve auxin response. *Plant Cell Environ.* 45, 871-883. doi:10.1111/pce.14137

Zhang, S., Li, Y.L., Song, G., Gao, J., Zhang, R., Li, W., et al. (2020). Heterologous expression of the *ThIPK2* gene enhances drought resistance of common wheat. *J. Integr. Agric.* 19, 941-952. doi:10.1016/S2095-3119(19)62714-0

Zhou, Y., Zhang, Y., Wang, X., Han, X., An, Y., Lin, S., et al. (2020). Root-specific NF-Y family transcription factor, *PdNF-YB21*, positively regulates root growth and drought resistance by abscisic acid-mediated indoylacetic acid transport in *Populus*. *New Phytol.* 227, 407-426. doi:10.1111/nph.16524
