# Supplementary material for: Responses of root system architecture to water stress at multiple levels: A meta-analysis of trials under controlled conditions
Source: Front Plant Sci. 2022 Dec 9;13:1085409. doi: 10.3389/fpls.2022.1085409 (PMC9780461; doi:10.3389/fpls.2022.1085409)
Supplement: Supplementary file 3 [file Table_3.docx]

***Supplementary File3. Reference-Root Trait***

Abdirad, S., Ghaffari, M.R., Majd, A., Irian, S., Soleymaniniya, A., Daryani, P., et al. (2022). Genome-wide expression analysis of root tips in contrasting rice genotypes revealed novel candidate genes for water stress adaptation. *Front. Plant Sci.* 13. doi:10.3389/fpls.2022.792079

Álvarez, S., and Jesús Sánchez-Blanco, M. (2013). Changes in growth rate, root morphology and water use efficiency of potted *Callistemon citrinus* plants in response to different levels of water deficit. *Sci. Hortic.* 156, 54-62. doi:10.1016/j.scienta.2013.03.024

Avramova, V., Nagel, K. A., AbdElgawad, H., Bustos, D., DuPlessis, M., Fiorani, F., et al. (2016). Screening for drought tolerance of maize hybrids by multi-scale analysis of root and shoot traits at the seedling stage. *J. Exp. Bot.* 67(8), 2453-2466. doi:10.1093/jxb/erw055

Bacher, H., Zhu, F., Gao, T., Liu, K., Dhatt, B. K., Awada, T., et al. (2021). Wild emmer introgression alters root-to-shoot growth dynamics in durum wheat in response to water stress. *Plant Physiol.* 187(3), 1149-1162. doi:10.1093/plphys/kiab292

Belachew, K.Y., Nagel, K.A., Fiorani, F., and Stoddard, F.L. (2018). Diversity in root growth responses to moisture deficit in young faba bean (*Vicia faba* L.) plants. *Peerj* 6. doi:10.7717/peerj.4401

Berny-Miery, Teran. J.C., Konzen, E.R., Medina, V., Palkovic, A., Ariani, A., Tsai, S.M., et al. (2019). Root and shoot variation in relation to potential intermittent drought adaptation of Mesoamerican wild common bean (*Phaseolus vulgaris* L.). *Ann. Bot.* 124 (6), 917-932. doi:10.1093/aob/mcy221

Boguszewska-Mankowska, D., Zarzynska, K., and Nosalewicz, A. (2020). Drought differentially affects root system size and architecture of potato cultivars with differing drought tolerance. *Am. J. Potato Res.* 97(1), 54-62. doi:10.1007/s12230-019-09755-2

Boudiar, R., Casas, A. M., Gioia, T., Fiorani, F., Nagel, K. A., and Igartua, E. (2020). Effects of low water availability on root placement and shoot development in landraces and modern barley cultivars. *Agronomy-Basel*. 10(1). doi:10.3390/agronomy10010134

Bristiel, P., Roumet, C., Violle, C., and Volaire, F. (2019). Coping with drought: root trait variability within the perennial grass Dactylis glomerata captures a trade-off between dehydration avoidance and dehydration tolerance. *Plant Soil.* 434(1-2), 327-342. doi:10.1007/s11104-018-3854-8

Chairi, F., Elazab, A., Sanchez-Bragado, R., Luis Araus, J., and Dolors Serret, M. (2016). Heterosis for water status in maize seedlings. *Agr. Water Manage.* 164, 100-109. doi:10.1016/j.agwat.2015.08.005

Chakhchar, A., Chaguer, N., Ferradous, A., Filali-Maltouf, A., and El Modafar, C. (2018). Root system response in *Argania spinosa* plants under drought stress and recovery. *Plant Signaling Behav.* 13(7). doi:10.1080/15592324.2018.1489669

Dayoub, E., Lamichhane, J. R., Schoving, C., Debaeke, P., and Maury, P. (2021). Early-stage phenotyping of root traits provides insights into the drought tolerance level of soybean cultivars. *Agronomy-Basel.* 11(1). doi:10.3390/agronomy11010188

Djanaguiraman, M., Prasad, P. V. V., Kumari, J., Sehgal, S. K., Friebe, B., Djalovic, I., et al. (2019a). Alien chromosome segment from *Aegilops speltoides* and *Dasypyrum villosum* increases drought tolerance in wheat via profuse and deep root system. *Bmc Plant Biol.* 19(242), (7 June 2019)-(2017 June 2019).

Djanaguiraman, M., Prasad, P. V. V., Kumari, J., and Rengel, Z. (2019b). Root length and root lipid composition contribute to drought tolerance of winter and spring wheat. *Plant Soil.* 439(1-2), 57-73. doi:10.1007/s11104-018-3794-3

Du, Y., Zhao, Q., Chen, L., Yao, X., Zhang, W., Zhang, B., and Xie, F. (2020). Effect of drought stress on sugar metabolism in leaves and roots of soybean seedlings. *Plant Physiol. Biochem.* 146, 1-12. doi:10.1016/j.plaphy.2019.11.003

Fadul, G. M. A., Wan, L., He, F., Xie, K., Qin, Y., and Li, X. (2016). Abscisic acid content in roots and root characteristics of alfalfa under deficit irrigation. *Afr. J. Agric. Res.* 11(11), 935-940.

Fort, F., Cruz, P., Catrice, O., Delbrut, A., Luzarreta, M., Stroia, C., et al. (2015). Root functional trait syndromes and plasticity drive the ability of grassland *Fabaceae* to tolerate water and phosphorus shortage. *Environ. Exp. Bot.* 110, 2-72. doi:10.1016/j.envexpbot.2014.09.007

Friedli, C. N., Abiven, S., Fossati, D., and Hund, A. (2019). Modern wheat semi-dwarfs root deep on demand: response of rooting depth to drought in a set of Swiss era wheats covering 100years of breeding. *Euphytica.* 215(4). doi:10.1007/s10681-019-2404-7

Früchtenicht, E., Neumann, L., Klein, N., Bonal, D., and Brueggemann, W. (2018). Response of *Quercus robur* and two potential climate change winners-*Quercus pubescens* and *Quercus ilex-*To two years summer drought in a semi-controlled competition study: I-Tree water status. *Environ. Exp. Bot.* 152, 107-117. doi:10.1016/j.envexpbot.2018.01.002

Gajanayake, B., Reddy, K. R., Shankle, M. W., and Arancibia, R. A. (2014). Growth, developmental, and physiological responses of two sweetpotato (*Ipomoea batatas* L. Lam ) cultivars to early season soil moisture deficit. *Sci. Hortic.* 168, 218-228. doi:10.1016/j.scienta.2014.01.018

Gebre, M. G., and Earl, H. J. (2021). Soil water deficit and fertilizer placement effects on root biomass distribution, soil water extraction, water use, yield, and yield components of soybean *glycine max* (L.) Merr. grown in 1-m rooting columns. *Fron. Plant Sci.* 12. doi:10.3389/fpls.2021.581127

Geng, D. L., Lu, L.Y., Yan, M.J., Shen, X.X., Jiang, L.J., Li, H.Y., et al. (2019). Physiological and transcriptomic analyses of roots from Malus sieversii under drought stress. *J. Integr. Agric.* 18(6), 1280-1294. doi:10.1016/s2095-3119(19)62571-2

Grašič, M., Golob, A., Vogel-Mikus, K., and Gaberscik, A. (2019). Severe water deficiency during the mid-vegetative and reproductive phase has little effect on Proso millet performance. *Water.* 11(10). doi:10.3390/w11102155

Grzesiak, M. T., Hordynska, N., Maksymowicz, A., Grzesiak, S., and Szechynska-Hebda, M. (2019). Variation among spring wheat (*Triticum aestivum* L.) genotypes in response to the drought stress. II-root system structure. *Plants-Basel.* 8(12). doi:10.3390/plants8120584

Haling, R. E., Brown, L. K., Bengough, A. G., Valentine, T. A., White, P. J., Young, I. M.,et al. (2014). Root hair length and rhizosheath mass depend on soil porosity, strength and water content in barley genotypes. *Planta.* 239(3), 643-651. doi:10.1007/s00425-013-2002-1

Hamedani, N.G., Gholamhoseini, M., Bazrafshan, F., Amiri, B., and Habibzadeh, F. (2020) Variability of root traits in sesame genotypes under different irrigation regimes. *Rhizosphere* 13. doi:10.1016/j.rhisph.2020.100190

Hammac, W. A., Pan, W. L., Bolton, R. P., and Koenig, R. T. (2011). High resolution imaging to assess oilseed species' root hair responses to soil water stress. *Plant Soil.* 39(1-2), 125-135. doi:10.1007/s11104-010-0335-0

Hashoum, H., Gavinet, J., Gauquelin, T., Baldy, V., Dupouyet, S., Fernandez, C., et al. (2021). Chemical interaction between Quercus pubescens and its companion species is not emphasized under drought stress. *Eur. J. For. Res.* 140(2), 333-343. doi:10.1007/s10342-020-01337-w

Hazman, M., and Brown, K. M. (2018). Progressive drought alters architectural and anatomical traits of rice roots. *Rice.* 11. doi:10.1186/s12284-018-0252-z

Henry, A., Cal, A.J., Batoto, T.C., Torres, R.O., and Serraj, R. (2012). Root attributes affecting water uptake of rice (*Oryza sativa*) under drought. *J. Exp. Bot.* 63 (13), 4751-4763. doi:10.1093/jxb/ers150

Hund, A., Ruta, N., and Liedgens, M. (2009). Rooting depth and water use efficiency of tropical maize inbred lines, differing in drought tolerance. *Plant Soil.* 318(1-2), 311-325. doi:10.1007/s11104-008-9843-6

Idrissi, O., Houasli, C., Udupa, S. M., De Keyser, E., Van Damme, P., and De Riek, J. (2015). Genetic variability for root and shoot traits in a lentil (*Lens culinaris* Medik.) recombinant inbred line population and their association with drought tolerance. *Euphytica.* 204(3), 693-709.doi:10.1007/s10681-015-1373-8

Kamphorst, S. H., do Amaral Junior, A. T., de Lima, V. J., Santos, P. H. A. D., Rodrigues, W. P., Vivas, J. M. S., et al. (2020). Comparison of selection traits for effective popcorn (*Zea mays* L. var. Everta) breeding under water limiting conditions. *Front. Plant Sci.* 11. doi:10.3389/fpls.2020.01289

Kartika, K., Sakagami, J.I., Lakitan, B., Yabuta, S., Wijaya, A., Kadir, S., et al. (2020). Morpho-physiological response of *Oryza glaberrima* to gradual soil drying. *Rice Sci.* 27(1), 67-74. doi:10.1016/j.rsci.2019.12.007

Kato, Y., Abe, J., Kamoshita, A., and Yamagishi, J. (2006). Genotypic variation in root growth angle in rice (*Oryza sativa* L.) and its association with deep root development in upland fields with different water regimes. *Plant Soil* 287 (1-2), 117-129. doi:10.1007/s11104-006-9008-4

Kerbiriou, P. J., Stomph, T. J., Van Der Putten, P. E. L., Van Bueren, E. T. L., and Struik, P. C. (2013). Shoot growth, root growth and resource capture under limiting water and N supply for two cultivars of lettuce (*Lactuca sativa* L.). *Plant Soil.* 371(1-2), 281-297. doi:10.1007/s11104-013-1672-6

Khalil, A. M., Murchie, E. H., and Mooney, S. J. (2020). Quantifying the influence of water deficit on root and shoot growth in wheat using X-ray Computed Tomography. *Aob Plants.* 12(5). doi:10.1093/aobpla/plaa036

Kulkarni, M., and Phalke, S. (2009). Evaluating variability of root size system and its constitutive traits in hot pepper (*Capsicum annum* L.) under water stress. *Sci. Hortic.* 120(2), 159-166. doi:10.1016/j.scienta.2008.10.007

Lahlou, O., and Ledent, J. F. (2005). Root mass and depth, stolons and roots formed on stolons in four cultivars of potato under water stress. *Eur. J. Agron.* 22(2), 159-173. doi:10.1016/j.eja.2004.02.004

Li, T., Yang, H., Zhang, W., Xu, D., Dong, Q., Wang, F.,et al. (2017). Comparative transcriptome analysis of root hairs proliferation induced by water deficiency in maize. *J. Plant Biol.* 60 (1), 26-34. doi:10.1007/s12374-016-0412-x

Liang, X., Erickson, J.E., Vermerris, W., Rowland, D.L., Sollenberger, L.E., and Silveira, M.L. (2017). Root architecture of sorghum genotypes differing in root angles under different water regimes. *J. Crop Improv.*31 (1), 39-55. doi:10.1080/15427528.2016.1258603

Liu, H. S., and Li, F. M. (2005). Root respiration, photosynthesis and grain yield of two spring wheat in response to soil drying. *Plant Growth Regul.* 46(3), 233-240. doi:10.1007/s10725-005-8806-7

Liu, T.Y., Chen, M.X., Zhang, Y., Zhu, F.Y., Liu, Y.G., Tian, Y., et al. (2019). Comparative metabolite profiling of two switchgrass ecotypes reveals differences in drought stress responses and rhizosheath weight. *Planta.* 250(4), 1355-1369. doi:10.1007/s00425-019-03228-w

Liu, T.Y., Ye, N., Song, T., Cao, Y., Gao, B., Zhang, D., et al. (2019). Rhizosheath formation and involvement in foxtail millet (*Setaria italica*) root growth under drought stress. *J. Integr. Plant Biol.* 61 (4), 449-462. doi:10.1111/jipb.12716

Lokhande, P. K., Naik, R. M., Dalvi, U. S., Mhase, L. B., and Harer, P. N. (2019). Antioxidative and root attributes response of chickpea parents and crosses under drought stress. *Legume Res.* 42(3), 320-325. doi:10.18805/lr-4031

MacAlister, D., Muasya, A. M., Crespo, O., Ogola, J. B. O., Maseko, S., Valentine, A. J., et al. (2020). Stress tolerant traits and root proliferation of *Aspalathus linearis* (Burm.f.) R. Dahlgren grown under differing moisture regimes and exposed to drought. *S. Afr. J. Bot.* 131, 342-350. doi:10.1016/j.sajb.2020.03.003

Maganti, M., Weaver, S., and Downs, M. (2005). Responses of spreading orach (*Atriplex patula*) and common lambsquarters (*Chenopodium album*) to soil compaction, drought, and waterlogging. *Weed Sci.* 53 (1), 90-96. doi:10.1614/ws-04-143r

Mishra, S. K., Khan, M. H., Misra, S., Dixit, V. K., Gupta, S., Tiwari, S., et al. (2020). Drought tolerant *Ochrobactrum* sp. inoculation performs multiple roles in maintaining the homeostasis in Zea mays L. subjected to deficit water stress. *Plant Physiol. Biochem.* 150, 1-14. doi:10.1016/j.plaphy.2020.02.025

Mo, Y., Yang, R., Liu, L., Gu, X., Yang, X., Wang, Y., et al. (2016). Growth, photosynthesis and adaptive responses of wild and domesticated watermelon genotypes to drought stress and subsequent re-watering. *Plant Growth Regul.* 79(2), 229-241. doi:10.1007/s10725-015-0128-9

Moles, T. M., Mariotti, L., Federico De Pedro, L., Guglielminetti, L., Picciarelli, P., and Scartazza, A. (2018). Drought induced changes of leaf-to-root relationships in two tomato genotypes. *Plant Physiol.* 128, 24-31. doi:10.1016/j.plaphy.2018.05.008

Moser, B., Kipfer, T., Richter, S., Egli, S., and Wohlgemuth, T. (2015). Drought resistance of *Pinus sylvestris* seedlings conferred by plastic root architecture rather than ectomycorrhizal colonisation. *Ann. Forest Sci.* 72(3). doi:10.1007/s13595-014-0380-6

Ouyang, W., Yin, X., Yang, J., and Struik, P. C. (2020). Comparisons with wheat reveal root anatomical and histochemical constraints of rice under water-deficit stress. *Plant Soil.* 452(1-2), 547-568. doi:10.1007/s11104-020-04581-6

Oyanagi, A., Sato, A., and Wada, M. (1992). Effect of water potential of culture medium on geotropic response of primary seminal root in Japanese wheat cultivars. *Jpn. J. Crop Sci.* 61(1), 119-123. doi:10.1626/jcs.61.119

Pang, J., Yang, J., Ward, P., Siddique, K. H. M., Lambers, H., Tibbett, M., et al. (2011). Contrasting responses to drought stress in herbaceous perennial legumes. *Plant Soil. 348*(1-2), 299-314. doi:10.1007/s11104-011-0904-x

Patel, D. S., Kirti, B., Dhiraji, P. P., Vipulkumar, P., Suchismita, J., Ajay, V. N., and Harshadkumar, N. C. (2021). Does plant root architecture respond to potassium under water stress? A case from rice seedling root responses. *Cur. Sci.* 120(6), 1050-1056. doi:10.18520/cs/v120/i6/1050-1056

Pires, M. V., de Castro, E. M., Morais de Freitas, B. S., Souza Lira, J. M., Magalhaes, P. C., and Pereira, M. P. (2020). Yield-related phenotypic traits of drought resistant maize genotypes. *Environ. Exp. Bot.* 171. doi:10.1016/j.envexpbot.2019.103962

Robin, A.H.K., Uddin, M.J., and Bayazid, K.N. (2015) Polyethylene glycol (PEG) treated hydroponic culture reduces length and diameter of root hairs of wheat varieties. *Agronomy*. 5, 506–518. doi:10.3390/agronomy5040506

Robin, A. H. K., Ghosh, S., and Abu Shahed, M. (2021). PEG-induced osmotic stress alters root morphology and root hair traits in wheat genotypes. *Plants-Basel.* 10(6). doi:10.3390/plants10061042

Rose, L., Leuschner, C., Koeckemann, B., and Buschmann, H. (2009). Are marginal beech (*Fagus sylvatica* L.) provenances a source for drought tolerant ecotypes? *Eur. J. Forest Res.* 128(4), 335-343. doi:10.1007/s10342-009-0268-4

Rostamza, M., Richards, R. A., and Watt, M. (2013). Response of millet and sorghum to a varying water supply around the primary and nodal roots. *Ann. Bot.* 112(2), 439-446. doi:10.1093/aob/mct099

Schneider, H. M., Lor, V. S. N., Hanlon, M. T., Perkins, A., Kaeppler, S. M., Borkar, A. N., et al. (2022). Root angle in maize influences nitrogen capture and is regulated by calcineurin B-like protein (CBL)-interacting serine/threonine-protein kinase 15 (*ZmCIPK15*). *Plant Cell Environ.* 45(3), 837-853. doi:10.1111/pce.14135

Sofi, P. A., Djanaguiraman, M., Siddique, K. H. M., and Prasad, P. V. V. (2018). Reproductive fitness in common bean (*Phaseolus vulgaris* L.) under drought stress is associated with root length and volume. *Indian J. Plant Physiol.* 23(4), 796-809. doi:10.1007/s40502-018-0429-x

Thangthong, N., Jogloy, S., Jongrungklang, N., Kvien, C.K., Pensuk, V., Kesmala, T., et al. (2018). Root distribution patterns of peanut genotypes with different drought resistance levels under early-season drought stress. *J. Agron. Crop Sci.* 204 (2), 111-122. doi:10.1111/jac.12249

Thangthong, N., Jogloy, S., Pensuk, V., Kesmala, T., and Vorasoot, N. (2016). Distribution patterns of peanut roots under different durations of early season drought stress. *Field Crops Res.*198, 40-49. doi:10.1016/j.fcr.2016.08.019

Trillana, N., Inamura, T., Chaudhary, R., and Horie, T. (2001). Comparison of root system development in two rice cultivars during stress recovery from drought and the plant traits for drought resistance. *Plant Prod. Sci.* 4(3), 155-159. doi:10.1626/pps.4.155

Tsuji, W., Inanaga, S., Araki, H., Morita, S., An, P., and Sonobe, K. (2005). Development and distribution of root system in two grain sorghum cultivars originated from Sudan under drought stress. *Plant Prod. Sci.* 8(5), 553-562. doi:10.1626/pps.8.553

Vanaja, M., Yadav, S. K., Archana, G., Lakshmi, N. J., Reddy, P. R. R., Vagheera, P., et al. (2011). Response of C-4 (maize) and C-3 (sunflower) crop plants to drought stress and enhanced carbon dioxide concentration. *Plant Soil Environ.* 57(5), 207-215. doi:10.17221/346/2010-pse

Vasellati, V., Oesterheld, M., Medan, D., and Loreti, J. (2001). Effects of flooding and drought on the anatomy of Paspalum dilatatum. *Ann. Bot.* 88(3). 355-360. doi:10.1006/anbo.2001.1469

Veerappa, R., Slocum, R. D., Siegenthaler, A., Wang, J., Clark, G., and Roux, S. J. (2019). Ectopic expression of a pea apyrase enhances root system architecture and drought survival in *Arabidopsis* and soybean. *Plant Cell Environ.* 42(1), 337-353. doi:10.1111/pce.13425

Vega Riveros. C., Villagra, P.E., and Greco, S.A. (2020). Different root strategies of perennial native grasses under two contrasting water availability conditions: implications for their spatial distribution in desert dunes. *Plant Ecol.* 221 (7), 633-646. doi:10.1007/s11258-020-01038-9

Wan, L., Li, Y., Li, S., and Li, X. (2022). Transcriptomic profiling revealed genes involved in response to drought stress in alfalfa. *J. Plant Growth Regul.* 41(1), 92-112. doi:10.1007/s00344-020-10287-x

Wang, X., Wang, Y., Wang, L., Liu, H., Zhang, B., Cao, Q., et al. (2018). *Arabidopsis* *PCaP2* functions as a linker between ABA and SA signals in plant water deficit tolerance. *Front. Plant Sci.* 9. doi:10.3389/fpls.2018.00578

Wang, Y., Peng, C., Zhan, Y., Yu, L., Li, M., Li, J., et al. (2017). Comparative proteomic analysis of two sugar beet cultivars with contrasting drought tolerance. *J. Plant Growth Regul.* 36(3), 537-549. doi:10.1007/s00344-017-9703-9

Wong, G. R., Mazumdar, P., Lau, S.-E., and Harikrishna, J. A. (2018). Ectopic expression of a *Musa acuminata* root hair defective 3 (*MaRHD3*) in *Arabidopsis* enhances drought tolerance. *J. Plant Physiol.* 231, 219-233. doi:10.1016/j.jplph.2018.09.018

Xiao, S., Liu, L., Zhang, Y., Sun, H., Zhang, K., Bai, Z., et al. (2020a). Fine root and root hair morphology of cotton under drought stress revealed with RhizoPot. *J. Agron. Crop Sci.* 206 (6), 679-693. doi:10.1111/jac.12429

Yang, Z., Cao, Y., Zhao, J., Zhou, B., Ge, X., Li, Q., et al. (2021). Root response of moso bamboo (*Phyllostachys edulis* (Carriere) J. Houz.) seedlings to drought with different intensities and durations. *Forests.* 12(1). doi:10.3390/f12010050

Ye, Z.Q., Wang, J.M., Wang, W.J., Zhang, T.H., and Li, J.W. (2019). Effects of root phenotypic changes on the deep rooting of *Populus euphratica* seedlings under drought stresses. *Peerj.* 7. doi:10.7717/peerj.6513

Zhang, F., Wang, P., Zou, Y.N., Wu, Q.S., and Kuca, K. (2019). Effects of mycorrhizal fungi on root-hair growth and hormone levels of taproot and lateral roots in trifoliate orange under drought stress. *Arch. Agron. Soil Sci.* 65(9), 1316-1330. doi:10.1080/03650340.2018.1563780

Zhou, Y., Zhang, Y., Wang, X., Han, X., An, Y., Lin, S., et al. (2020). Root-specific NF-Y family transcription factor, *PdNF-YB21*, positively regulates root growth and drought resistance by abscisic acid-mediated indoylacetic acid transport in *Populus*. *New Phytol.* 227 (2), 407-426. doi:10.1111/nph.16524
